# Self-Assembled Microplastic-Free Microcapsules Using Aromatic Bis-Ureas with Improved Strength and Tunable Barrier Properties for Encapsulating Cinmethylin

**DOI:** 10.1021/acsami.5c06238

**Published:** 2025-05-14

**Authors:** Siddhant Pravin Bhutkar, Pierre-Eric Millard, Henning Urch, Jon A. Preece, Zhibing Zhang

**Affiliations:** † School of Chemical Engineering, 1724 University of Birmingham, Birmingham B15 2TT, U.K.; ‡ School of Chemistry, 1724 University of Birmingham, Birmingham B15 2TT, U.K.; § 5184 BASF SE, 67056 Ludwigshafen am Rhein, Germany; ∥ BASF SE, 67117 Limburgerhof, Germany

**Keywords:** microplastic free bis-urea, microcapsules, agrochemical, controlled release, self-assembly

## Abstract

Microencapsulation technology can be used for safe handling and controlled release of agrochemicals. Commercial microencapsulated formulations typically use cross-linked polymeric microcapsules, which encapsulate agrochemicals for improved efficiency and precise application. However, these polymeric microcapsules are nonbiodegradable and add to the growing microplastic pollution challenge at the end of their life cycle. Herein, we demonstrate a simple one-pot process for the interfacial self-assembly of aromatic bis-urea molecules to synthesize microplastic-free microcapsules encapsulating cinmethylin, an effective cineolic pre-emergence herbicide commonly used against grass weeds in annual crops. The urea linkages act as hydrogen-bonding motifs forming a self-assembled supramolecular shell at the oil–water interface. The shell material’s chemical composition was analyzed using infrared spectroscopy, ^1^H-NMR, and mass spectrometry. Four batches of well-dispersed microcapsules (diameter, 1–10 μm) with encapsulation efficiency >99% and varying payload were synthesized. Accelerated thermal release tests proved that encapsulation reduced the cinmethylin evaporation by up to 90%, over nonencapsulated cinmethylin, and crucially, the release profiles of the bis-urea microcapsules were comparable to conventional polyurea microcapsules prepared industrially. The release rate of cinmethylin increased with payload, indicating that barrier properties of bis-urea microcapsules are tunable, making them adaptable for encapsulating a variety active ingredients. Additionally, all the four batches of bis-urea microcapsules were mechanically stronger than the polyurea microcapsules. Synthesized using a straightforward process requiring no modifications to existing industrial equipment, these bis-urea microcapsules have great potential to replace commercial nonbiodegradable microplastic microcapsules.

## Graphical abstract

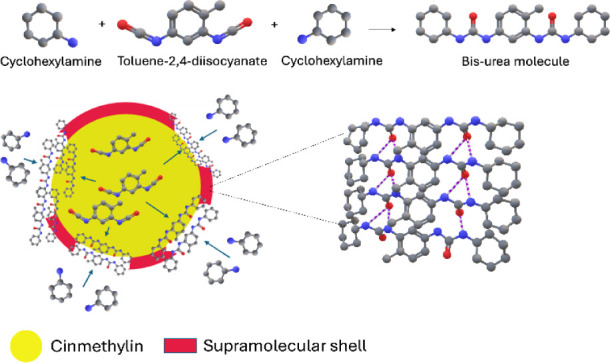

## Introduction

The effective use of agrochemicals to increase agricultural yield is crucial for meeting rising global food demands.^1^ Conventionally, agrochemicals can be divided into four major categories: pesticides, fertilizers, plant growth regulators and soil conditioning chemicals.^2^ These chemicals play a key role in improving the quality and quantity of crop production worldwide. Consequently, the agrochemical market is expected to be worth $300 Bn by 2025.^3^ Nevertheless, the adverse impact agrochemicals have on the natural environment and human health is widely known.^3–5^ Controlled release formulations (CRFs) are gaining interest for overcoming the environmental problems related to agrochemicals by controlling dosage, reducing the number of applications through sustained release, protecting the active ingredient from the external environment and reducing the environmental pollution by reducing excessive application of the agrochemicals.^6,7^

Microencapsulation technology is now well-established in the agrochemical sector for developing polymeric CRFs.^8^ Microencapsulation processes typically involve isolation of active ingredients (AI) by enveloping in an external shell for stabilization and controlled release at the application site.^9^ Polymeric microcapsules have gained interest in commercial applications owing to their ease of synthesis and effective barrier properties.^7^ Interfacial polymerization is commonly used for synthesizing microcapsules using synthetic polymers like polyurea, polyurethane, and polyamide.^10–13^ A variety of agrochemicals including herbicides, insecticides and fertilizers have been encapsulated using interfacial polymerization.^1,14–17^ However, despite their superior performance, the nonbiodegradability of these polymeric microcapsules is a cause for concern as they are adding to the microplastic pollution challenge, at the end of their life cycle.^18–20^

The alarming rise in microplastic pollution is now a major problem globally.^21^ The accumulation of microplastic pollutants can have the potential to adversely affect both, biodiversity and soil fertility.^19,22^ Consequently, intentionally added microplastics will be restricted from certain applications in Europe as per the regulations set out by the European Chemicals Agency (ECHA).^23^ Therefore, finding microplastic-free alternatives for encapsulating AI becomes critical for businesses and sustainable development. However, simply designing microplastic-free microcapsules is not sufficient. The synthesis procedure employed must be simple, safe and robust to be industrially viable. Furthermore, the resultant microcapsules must have adjustable mechanical and barrier properties with the AI release performance at par with the microcapsules employed currently in commercial formulations. Lobel et al. recently reviewed these challenges in developing sustainable microencapsulation procedures which can effectively replace the current nonbiodegradable formulations.^20^

Cinmethylin is an ether derivative of the cineole family, which acts as an effective pre-emergence herbicide against grass weeds for a variety of annual crops.^24–26^ Commercially developed microcapsules encapsulating cinmethylin are designed to improve its efficacy by improving its compatibility with other components in herbicidal formulations and providing a controlled release upon application.^27,28^ These commercial microcapsules are prepared using polyurea/polyurethane shell materials and have proven effective as CRFs for a variety of agrochemicals by minimizing environmental losses of the AI upon application.^29–31^ However, as mentioned hitherto, these polymeric microcapsules are nonbiodegradable.

In our recent work, a novel method to design microplastic-free microcapsules using supramolecular self-assembly of small bis-urea molecules was demonstrated.^32^ Here, the microcapsule shell is formed solely as a result of supramolecular hydrogen bonding between the bis-urea molecules in a simple, one-pot *in situ* process. The mechanical strength of these bis-urea microcapsules was comparable to certain commercial polymeric microcapsules, but there is potential for further improvement to enhance their competitiveness. Furthermore, a direct comparison in terms of controlled release performance of these bis-urea microcapsules with polymeric microcapsules was not explored.

Building upon the constraints identified in previous findings, this work highlights the use of aromatic bis-urea molecules for synthesizing microplastic-free microcapsules. Using cinmethylin as a model active ingredient, the release performance was tuned by varying the shell percentage in the capsules. Furthermore, the impact of including a central aromatic ring in the bis-urea molecule, relative to an aliphatic carbon chain,^32^ on the mechanical strength of the resultant microcapsules was studied. Most importantly, the performance of these novel microplastic-free microcapsules, in terms of the controlled release of cinmethylin, was compared with conventional polyurea microcapsules using an isothermal thermogravimetric analysis. To the best of our knowledge, aromatic bis-urea molecules have never been investigated for encapsulating AI. The results of this work demonstrate that these newly developed eco-friendly microcapsules have great potential to replace conventional nonbiodegradable polymeric microcapsules.

## Experimental Section

### Materials

Poly­(vinyl alcohol) (PVA, commercial name Mowiol 40–88, average *M*_w_ ∼ 205 000 g/mol, 88% hydrolyzed), cinmethylin (technical grade) and cinmethylin microcapsules were procured from BASF SE (Limburgerhof, Germany). Toluene-2,4-diisocyanate (TDI, technical grade 80%), cyclohexylamine, acetonitrile (HPLC grade), water (HPLC grade), dichloromethane (DCM), and dimethyl sulfoxide (DMSO) were procured from Merck (Dorset, UK). All the chemicals were used as received. Double distilled water was used for all experiments except for the high-pressure liquid chromatography (HPLC), where HPLC grade was used.

### Microcapsule Synthesis

An aqueous stock solution of PVA (100 g, 10% w/w) was prepared in advance. PVA (10 g) was gradually added to deionized water (90 g) under continuous stirring at 200 rpm using a magnetic stirrer hot plate (IKA RCT digital, IKA-Werke GmbH, Germany) equipped with a Proportional-Integral-Derivative (PID) temperature controller. The temperature was increased to 85 °C and maintained for 30 min to ensure complete dissolution of the polymer. Heating was then discontinued, and the solution was allowed to cool to room temperature (25 °C). This PVA stock solution was diluted as required and used consistently across all experiments to minimize batch-to-batch variability.

The microcapsules were synthesized using the procedure developed by Bhutkar et al.^32^ with modifications. Briefly, an aqueous solution of PVA (15.6 g, 10% w/w) was diluted with water (54.6 g) to make a homogeneous continuous phase. This continuous phase was transferred to a 250 mL double glazed jacketed reactor maintained at 12 °C using an external water bath (Julabo F33-HL, Germany). 1-Octanol (∼20 mg) was added to the continuous phase as a defoamer. Separately, TDI (3.65 g) was mixed with cinmethylin (31.2 g) to make a homogeneous oil phase. This oil phase was added to the continuous phase and emulsified using a Silverson L4RT homogenizer with an emulsor screen at a speed of 6000 rpm for 2 min. After the emulsification, four baffles were placed inside the reactor diametrically opposite each other. The Silverson homogenizer was replaced with an overhead stirrer (IKA Eurostar 20, Germany) attached to a Rushton turbine impeller and the emulsion was stirred at 500 rpm. A solution of cyclohexylamine (4.15 g) in water (26.6 g) was added to the emulsion over 15 min (flow rate: 2.1 mL min^–1^) using a syringe pump (Harvard Apparatus, Pump 11 Elite). The temperature was maintained at 12 °C during the addition. After the addition, the temperature was raised to 60 °C at 1 K min^–1^ and maintained at 60 °C for 3 h. The resultant microcapsule dispersion was then cooled to 20 °C and stored at room temperature for further analysis.

Four batches of microcapsules were prepared, each with different cinmethylin content and shell percentages. The shell percentage represents the weight fraction of the capsule’s shell relative to the total capsule weight, calculated as

1 $$\mathrm{shell}\;\mathrm{percentage}\;\left(\mathrm{wt}\;\%\right)=\frac{\mathrm{mass}\;\mathrm{of}\;\;\mathrm{shell}\;\mathrm{components}\;\left(\mathrm{TDI}+\mathrm{cyclohexylamine}\right)}{\mathrm{total}\;\mathrm{capsule}\;\mathrm{mass}\;\left(\mathrm{cinmethylin}+\mathrm{shell}\;\mathrm{components}\right)}\times 100$$

The amounts of cinmethylin, TDI, and cyclohexylamine used in each batch are listed in Table 1. The total theoretical mass of the capsules was determined by summing the weights of cinmethylin and the shell components (TDI and cyclohexylamine). For each batch, during amine addition, the flow rate of the syringe pump was adjusted after calibration to complete the addition in 15 min. The shell percentage will be used to refer to these four batches of microcapsules henceforth.

**Table 1:** Mass of Cinmethylin, Cyclohexylamine, and TDI in the Four Prepared Baches of Microcapsules

Shell percentage (wt %)	Cinmethylin (g)	Total shell (g)	Cyclohexylamine (g)	TDI (g)
20	31.2	7.80	4.15	3.65
15	33.2	5.85	3.12	2.74
10	35.1	3.90	2.08	1.82
5	37.1	1.95	1.04	0.91

### Preparing Pure Bis-Urea Standard

To prepare the pure bis-urea standard, a solution of cyclohexylamine (2.27 g, 0.0228 mol) in chloroform (150 mL) was transferred to a jacketed reactor (250 mL) and stirred using an overhead stirrer (150 rpm, IKA Eurostar 20, Germany) equipped with a Rushton turbine type impeller. A solution of TDI (2 g, 0.0114 mol) in chloroform (3.5 mL) was added dropwise to the amine solution and the temperature was increased at 1 K min^–1^ to 45 °C and maintained for 2 h. The cyclohexylamine reacts rapidly with the TDI and the bis-urea begins to precipitate out as a white solid. At the end of 2 h, the temperature of the reactor was reduced to 20 °C and the precipitate was filtered out using Whatman filter paper (pore size ∼ 2 μm, grade 6, Cytiva, UK) attached to a Buchner funnel and a conical flask with a Laboport N938 vacuum pump (KNF, Germany). The solids collected were air-dried overnight at room temperature in a fume hood to obtain powdered bis-urea. Scheme 1 represents this bis-urea formation.

**Scheme 1:**
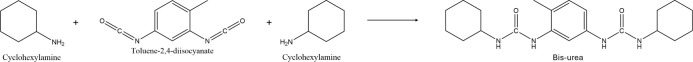
Chemical Reaction for Bis-Urea Formation

### Preparing Empty Capsules for Analysis

The procedure described above for fabricating cinmethylin microcapsules was repeated by replacing the cinmethylin (31.2 g) with DCM (31.2 g). At the end of the experiment, the capsule dispersion obtained was filtered and dried in an oven (40 °C, ∼18 h) to evaporate the DCM. The dry powder obtained was stored for characterization.

### Fourier Transform Infrared (FT-IR) Spectroscopy

To characterize the pure bis-urea and the microcapsule shell material chemically, the dry powder of both materials was analyzed using a Tensor 27 FT-IR spectrometer (Bruker USA) coupled with an attenuated total reflection (ATR) module. For testing, the dry powdered sample (∼20–30 mg) was placed in the sample holder and the spectrum was recorded from 4000 to 400 cm^–1^ (resolution of 3 cm^–1^). Each spectrum was an average of 200 scans.

### Bright-Field Microscopy

Optical images were recorded using a Motic AE31E microscope equipped with a Leica DFC7000 T camera which was linked to a computer using Leica Application Suite X imaging software for capturing images.

### Particle Size Distribution

A Malvern Mastersizer 2000 (Malvern Instruments Ltd., UK) was used for measuring the size of the cinmethylin microcapsules. For measurement, ca. 50–100 μL of the microcapsule dispersion obtained after synthesis was added to the aqueous dispersion unit of the instrument containing water (150 mL) and stirred (1500 rpm). A He–Ne laser operating at room temperature measured the size of the microcapsules using laser diffraction. A universal model based on the Mie theory provided by the supplier was used for data analysis.

### Scanning Electron Microscopy (SEM)

For studying the morphology of the microcapsules, the microcapsule dispersion after the reaction was diluted with water (∼1:30 w/w). An adhesive carbon tape (Agar Scientific, UK) was attached to an aluminum SEM stub. A drop of the diluted microcapsule slurry was placed on the carbon tape and air-dried (18 h) at room temperature. Prior to imaging, the capsules were coated with a thin layer (∼8 nm) of platinum using a Polaron Sputter Coater SC7640 (QuorumTech, UK). The stub was then mounted onto a FEI Quanta 3D Dual Beam (FEI GmbH, Germany) Field Emission Gun (FEG) microscope for imaging. The electron beam operated at 15 kV (0.8 pA current) with a working distance of 10 mm and the vacuum inside the SEM chamber was ∼10^–4^ mbar.

### High Pressure Liquid Chromatography (HPLC)

The quantification of cinmethylin in microcapsules was carried out using a Thermo Vanquish HPLC (Thermo Scientific, Germany) system equipped with a UV detector and an autosampler. A Syncronis C18 column (Thermo Scientific, Germany, 250 mm × 4.6 mm) was calibrated for cinmethylin using aqueous acetonitrile (65 wt %) carrier solvent with an isocratic gradient. For each run, the column temperature was maintained at 40 °C and the UV signal was measured at 208 nm. The carrier solvent flow rate was maintained at 1 mL min^–1^ with an injection volume of 20 μL for each sample. Pure cinmethylin had a retention time of 16 min and the run time for each sample was 30 min.

### Payload and Encapsulation Efficiency (EE)

The microcapsule dispersion (1 g) obtained after the reaction was added to water (9 g) and dispersed using a vortex mixer (IKA Genius 3) for 3 min. This diluted dispersion was filtered using a Whatman filter paper (pore size ∼ 2 μm, grade 6, Cytiva, UK) attached to a Buchner funnel and a conical flask with a Laboport N938 vacuum pump (KNF, Germany). The residual microcapsules collected on the filter paper were dried in an oven (40 °C for 18 h) and the clear filtrate was analyzed using the HPLC to calculate the amount of unencapsulated cinmethylin.

The dry residual microcapsules (∼100 mg) collected on the filter paper were recovered and added to aqueous acetonitrile (65% wt, 200 g) in a Duran flask (250 mL) with a screw cap, and ultrasonicated for 2 h to extract the cinmethylin inside the capsules into the solution. The aqueous acetonitrile was then filtered using a 0.22 μm syringe filter and the amount of cinmethylin was analyzed using HPLC. Subsequently, the payload was estimated as

2 $$\mathrm{payload}\;\left(\%\right)=\frac{{\mathrm{mass}}_{\mathrm{cin}}}{{\mathrm{mass}}_{\mathrm{capsules}}}\times 100$$

wherein, mass_cin_ is the mass of cinmethylin extracted into the acetonitrile and mass_capsules_ is the total mass of dry microcapsules used in the experiment.

For quantifying the total cinmethylin, the microcapsule dispersion (250 mg) obtained after the reaction, was added to aqueous acetonitrile (65% wt, 100 g) in a Duran flask with a screw cap (150 mL). The solution was then ultrasonicated using an ultrasound water bath (VWR International, Belgium) for 2 h to extract the cinmethylin from the microcapsules into the solvent. The acetonitrile solution was filtered after sonication using a 0.22 μm syringe filter and the amount of cinmethylin extracted (*W*_extract_) was analyzed using the HPLC. Since 250 mg of the microcapsule dispersion was used for the test, the total amount of cinmethylin in 1 g of reaction mixture (*W*_total_) was calculated as

3 $$W_{\mathrm{total}}=4\times W_{\mathrm{extract}}$$

and

4 $$\mathrm{EE}\;\left(\%\right)=\frac{W_{\mathrm{total}}-W_{1}}{W_{\mathrm{total}}}\times 100$$

where *W*_1_ represents the unencapsulated cinmethylin recovered in the clear filtrate. Both the experiments for payload and EE were performed in duplicates.

### Mechanical Strength of the Microcapsules

The mechanical strength of the microcapsules was determined by diametrically compressing individual capsules between two parallel surfaces. The micromanipulation rig developed by Zhang et al.^33^ was used for this purpose as demonstrated by Bhutkar et al.^32^ The microcapsule dispersion was significantly diluted (∼1:1000 w/w) with water, drop-casted onto a glass slide (∼2 cm × 1 cm) and dried overnight at room temperature. The slide was mounted onto the micromanipulator stage placed below a glass probe with a flat tip 70 μm in diameter connected to a force transducer (403*A*/1529011, Aurora Scientific Inc. Canada). A servo motor (DC Servocontroller, CONEX-C, USA) was used to control the upward and downward movement of the probe accurately (±0.2 μm) at a speed of 2 μm s^–1^. Thirty individual capsules tabbed randomly were ruptured. The force transducer was calibrated to record its sensitivity (0.4939 mN V^–1^) for converting its output voltage to force.^32^

### Thermal Analysis

To estimate the impact of encapsulation on the controlled release of cinmethylin, the dry microcapsules were exposed to a constant temperature (130 °C) and the weight loss was measured using thermogravimetric analysis (TGA). First, the microcapsule dispersion was diluted (1:50 w/w) with water and mixed on a vortex mixer and then centrifuged (SIGMA, 2–16 KL, Germany, EU) at 5000 rpm (23.2 N) for 5 min to allow the capsules to settle. The clear supernatant was discarded, and the washing and centrifuge cycle was repeated twice to remove excess PVA surfactant. The wet microcapsules, settled in the centrifuge tube were frozen at −20 °C overnight for 18h and then freeze-dried using SCANVAC–COOLSAFE Freeze drier (Labogene, Denmark) at −51 °C under vacuum (∼0.5 mbar) to get dry powdered microcapsules. These dry microcapsules obtained were analyzed using the Simultaneous Thermal Analyzer (STA) 449 F5 (Netsch, UK). The capsules (∼10 mg) were placed in an aluminum pan and loaded into the STA furnace. The temperature of the furnace was maintained at 130 °C for 3 h and the weight loss from the samples was recorded. The same protocol was followed to test the polyurea microcapsules. As a control for comparison, the experiment was also conducted using pure unencapsulated cinmethylin (∼10 mg).

In a separate experiment, the dry microcapsule shell material was exposed to a temperature ramp from 25 to 500 °C (10 K min^–1^) in the STA and the melting point was recorded using differential scanning calorimetry (DSC).

## Results and Discussion

### Reaction Chemistry

The self-assembly of bis-urea molecules at the oil–water interface can be tuned effectively by changing the isocyanate and amine used for synthesis which consequently affects the resultant microcapsule properties.^32^ In this work, a toluyl aromatic moiety was introduced in the bis-urea molecule using TDI to synthesize the microcapsules. Figure 1 shows the schematic representation of the microcapsule formation process. While fabricating the microcapsules, TDI was mixed with the cinmethylin to form a homogeneous oil phase which was emulsified into an aqueous solution of PVA to form a stable oil-in-water emulsion. After addition of cyclohexylamine solution, the bis-urea forms (Scheme 1) and self-assembles at the interface to generate the supramolecular shell by virtue of hydrogen bonds. During the synthesis, the ratio between the −NCO and −NH_2_ functional groups was fixed to 1. Since the cyclohexylamine has only one reactive group, the formation of a long-chain covalent polymer was not possible, thus avoiding the formation of the conventional polyurea microplastic structure. Whereas each bis-urea molecule has two urea linkages to act as two hydrogen bonding units leading to a noncovalent supramolecular polymer, which is inherently not a microplastic.

**Figure 1:**
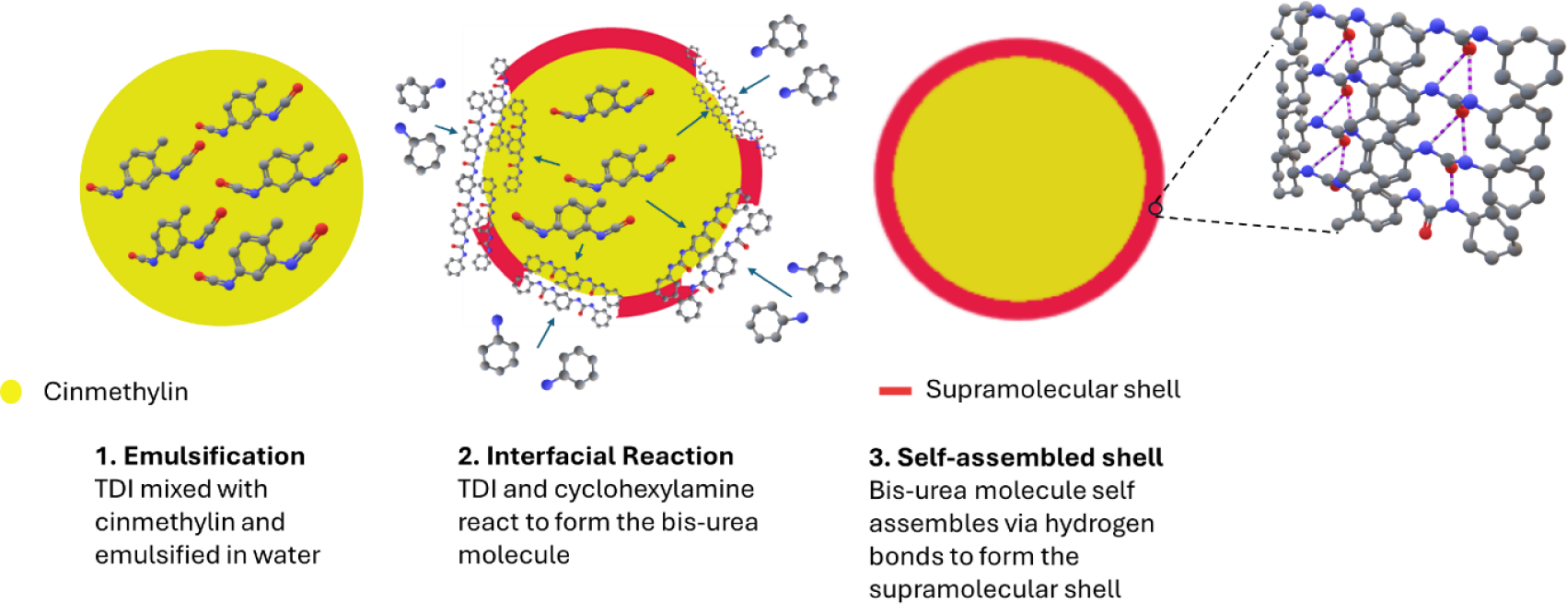
Interfacial formation and self-assembly of the bis-urea shell.

### Chemical Composition of the Shell Material

The bis-urea molecule was prepared by reacting the TDI with cyclohexylamine in chloroform as per Scheme 1. In this nonaqueous solvent, the reaction yields no side-products and the pure bis-urea molecule, which precipitates out, was used as a standard for chemical composition. During the microcapsule fabrication, the TDI is present in the dispersed oil phase and the cyclohexylamine is dissolved in the continuous aqueous phase. The subsequent bis-urea formation takes place at the interface (see Figure 1). However, the −NCO groups of the TDI have the potential to hydrolyze to −NH_2_ functionalities. Scheme 2 represents one such example wherein one −NCO group of the TDI molecule reacts with the cyclohexylamine and the other hydrolyzes to the amine, which has the potential to react with an −NCO group from another TDI, leading to the formation of oligomeric side products with additional urea linkages.

**Scheme 2:**
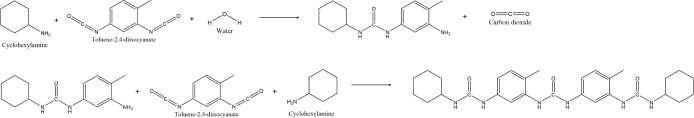
Formation of Side-Product with Three Urea Linkages

Therefore, to determine the exact chemical composition of the shell material, the microcapsule fabrication process was repeated by replacing cinmethylin with DCM as a substitute which could be removed easily after microcapsule formation and allow shell characterization. All other reaction conditions were kept identical to the actual microcapsule synthesis. Subsequently, the DCM was evaporated to yield the microcapsule shell material fabricated in aqueous conditions. Both the pure standard bis-urea and the empty microcapsule shell material were analyzed by mass spectrometry (Section S1), ^1^H NMR (Section S2), and FTIR (Figure 2).

**Figure 2:**
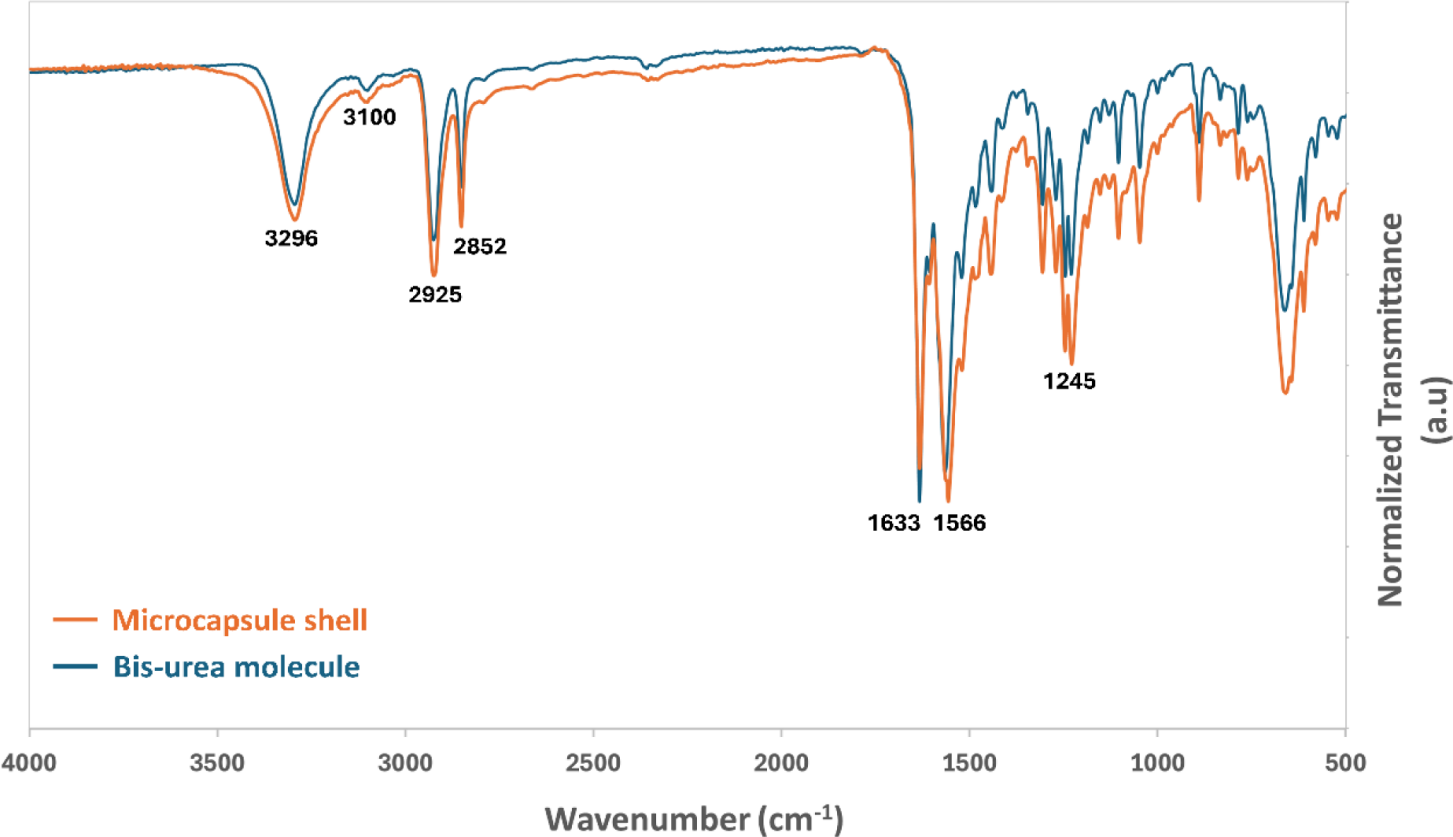
FTIR spectra of the microcapsule shell superimposed with the bis-urea molecule.

The mass spectrometry revealed that the masses obtained were identical (373 g mol^–1^) and associated with the bis-urea (Scheme 1), irrespective of synthetic route. Additionally, looking at higher molecular weights (up to *m*/*z* = 2500 g mol^–1^), there was no evidence of oligomer formation such as that proposed in Scheme 2 (520 g mol^–1^). Furthermore, comparison of CDCl_3_ solutions analyzed by ^1^H NMR spectra for the bis-urea molecule and the microcapsule shell revealed they were identical (Figure S2) and in agreement with proton assignments and peak integrations for the bis-urea structure (Scheme 1).

Finally, the FT-IR analysis is shown in Figure 2 of both the microcapsule shell material (orange line) alongside the pure bis-urea molecule (blue line). As can be seen the two spectra are very similar with strong peaks at 3296 cm^–1^ and 1633 cm^–1^ indicative of the −NH and carbonyl stretching of the urea moiety, respectively.^34,35^ The free −NH and urea carbonyl groups usually tend to exhibit absorption around 3420 cm^–1^ and 1700 cm^–1^, respectively. However, the strong hydrogen bonding results in a shift toward lower wavenumbers for both these groups.^36,37^ The absorption band at 1566 cm^–1^ corresponds to the -NH plane bending vibrations and the one at 1245 cm^–1^ represents the C–N stretching.^34^ The aliphatic C–H stretching vibrations typically have an absorption range from 2840 to 3000 cm^–1^. The two peaks at 2852 and 2925 cm^–1^ therefore correspond to the saturated C–H groups in the cyclohexane ring. The weak peak at 3100 cm^–1^ represents the aromatic C–H stretching from the central benzene ring.

Taken together, these three analytical techniques suggest that the reaction of the TDI with cyclohexylamine in the emulsion leads only to the desired simple microplastic-free bis-urea, and no higher oligomeric structures. Additionally, the inherent reaction kinetics favor the reaction of the isocyanate with the amine over hydrolysis, as the relative reaction rate of −NCO with −NH_2_ is almost 1000 times higher than with water under ambient conditions.^38^ Furthermore, interfacial reactions of isocyanates and amines forming microcapsules are known to be fast, reaching completion within seconds (200–800 s, 60 °C).^39^ Therefore, formation of the desired bis-urea molecule is also favored kinetically over the undesired oligomers.

### Shell Percentage of the Microcapsules

Commercially, cinmethylin microcapsules are designed to enhance the compatibility of cinmethylin with other herbicides in a formulation and to achieve controlled release without compromising its herbicidal activity.^40^ To achieve this optimum release performance, the shell percentage of the cinmethylin microcapsules is varied, typically between 1 and 20% (wt %).^27,29,40^ To check if the microcapsules prepared using the supramolecular bis-urea shell can be tuned in a similar fashion, four different batches of microcapsules were fabricated with decreasing shell percentage as described in Table 1. Figure 3 shows the optical images of the microcapsule dispersion for each batch. Well dispersed capsules of roughly 1 to 10 μm in diameter were observed without any aggregation.

**Figure 3:**
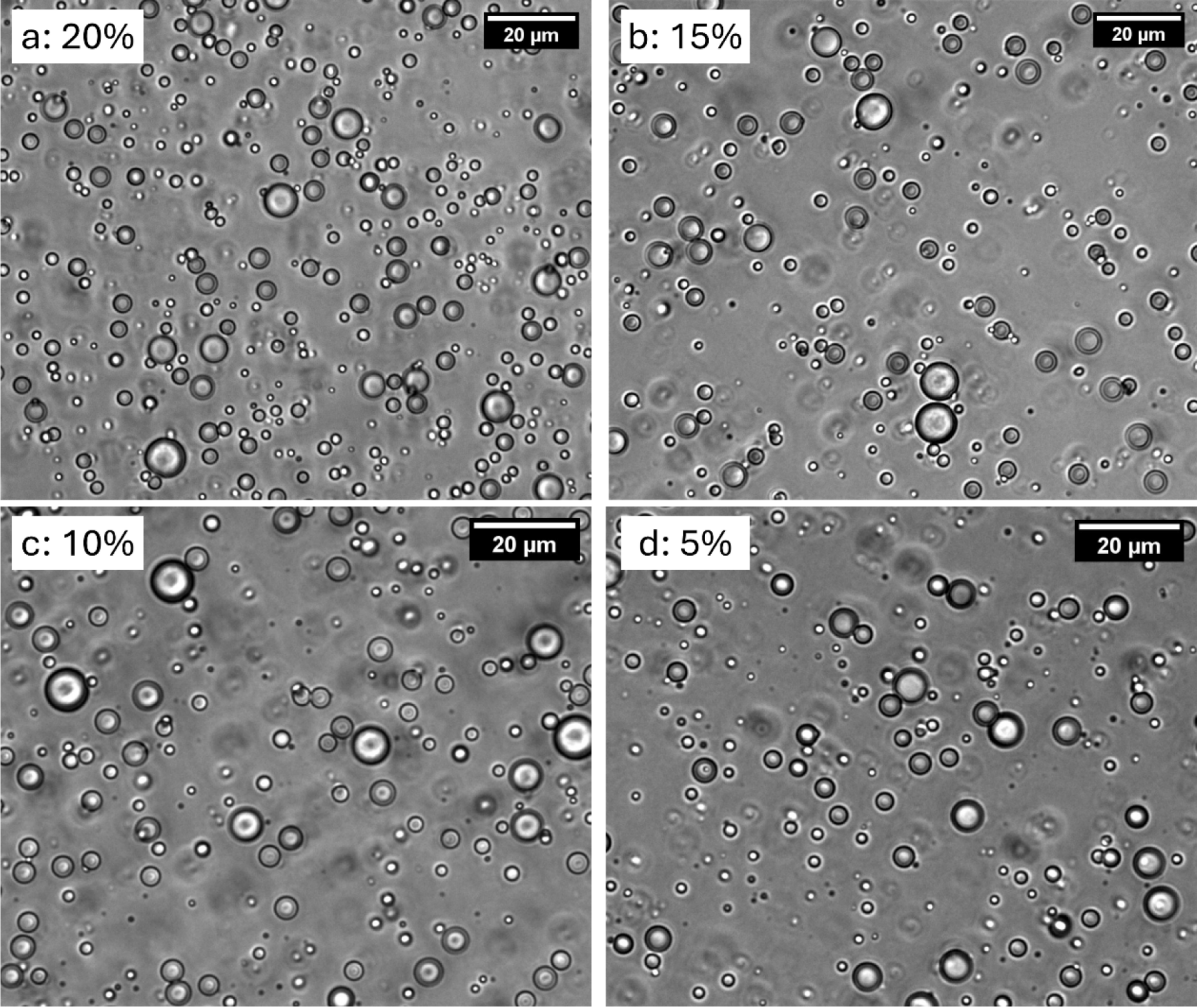
Optical images of microcapsules dispersion with shell percentage (a) 20% (b) 15% (c) 10% and (d) 5%.

Figure 4 shows the particle size distribution (PSD) for each batch. All four batches had a PSD with the mean Sauter diameter (*d*[3,2]) of roughly 2 μm. Microcapsules with 10% shell had a maximum SPAN of 2.5. These values are typical for emulsions produced using rotor-stator assemblies like the Silverson homogenizer used in this work.^41^ Moreover, well dispersed microcapsules without any aggregation, measuring 1–10 μm in size were realized. This size range is essential for agrochemical formulations which are sprayed onto the fields during application to avoid blocking the spray nozzles.^27^

**Figure 4:**
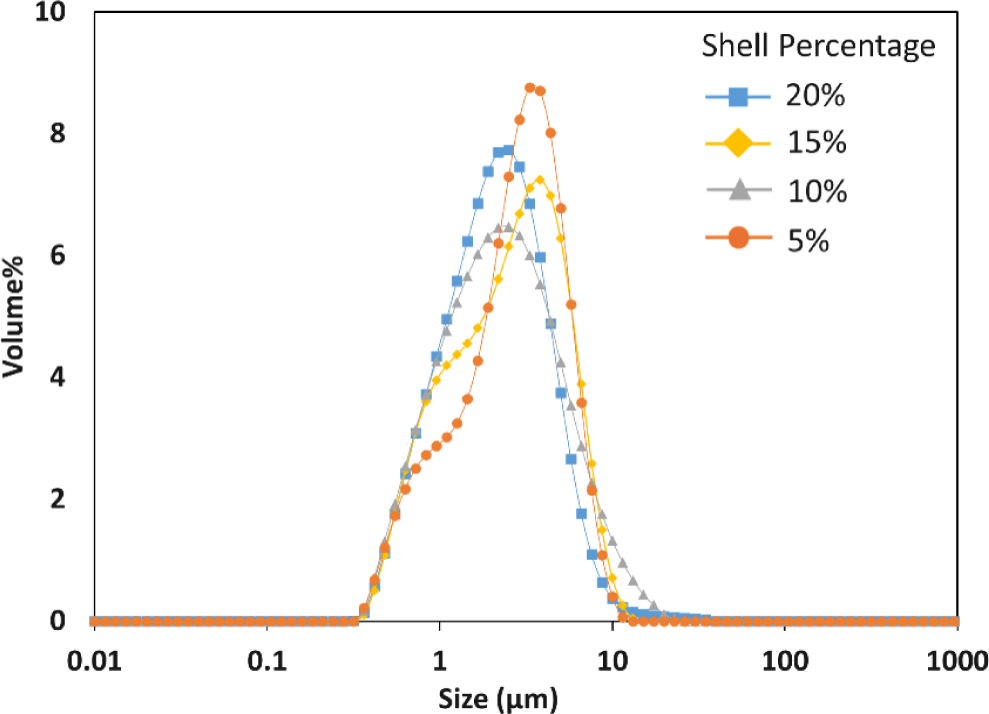
Particle size distribution for all four batches of microcapsules.

### Morphology

To study the structure and morphology of the shell, microcapsules were observed using SEM (Figure 5). Microcapsules with 20% shell were spherical and well formed (Figure 5a). However, as the shell percentage decreased from 15% to 5%, the capsules started to deform. At 15% shell (Figure 5b), some of the microcapsules were deformed with a wrinkled shell. The number of deformed capsules increased at 10% shell (Figure 5c) and subsequently, most of the microcapsules with 5% shell (Figure 5d) were damaged. Originally, in a wet dispersion, all the microcapsules appeared to be spherical (Figure 3). Therefore, the deformation of the shell structure was most likely caused by the high vacuum conditions (∼10^–4^ mbar) prevalent in the SEM chamber and due to the irradiation by the high voltage electron beam (∼15 kV). Furthermore, the increase in deformation with the lowering of shell percentage suggested that the mechanical strength of the microcapsules also decreased with shell percentage. Figure 6 shows some of the broken microcapsules observed at 5% shell, highlighting the core–shell structure with an oil-hosting cavity. The shell thickness of these microcapsules is estimated to be approximately 400 nm; however, precise quantification via SEM is limited due to challenges in obtaining clean cross sections. Future studies employing transmission electron microscopy (TEM) will enable accurate, statistical analysis of shell thickness. Correlating these measurements with shell percentage would offer valuable insight into shell formation.

**Figure 5:**
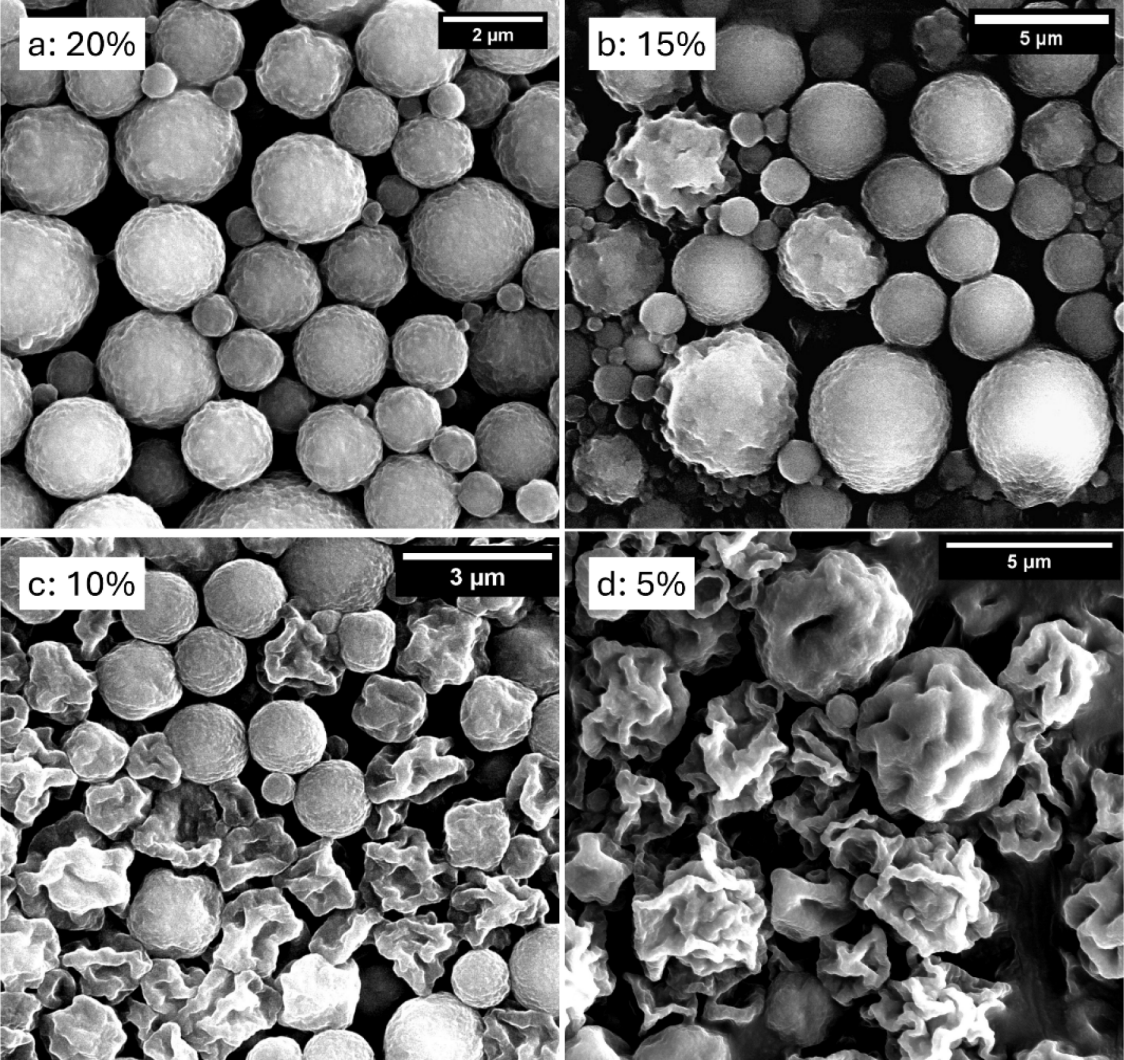
SEM images of microcapsules with shell percentage (a) 20% (b) 15% (c) 10% and (d) 5%.

**Figure 6:**
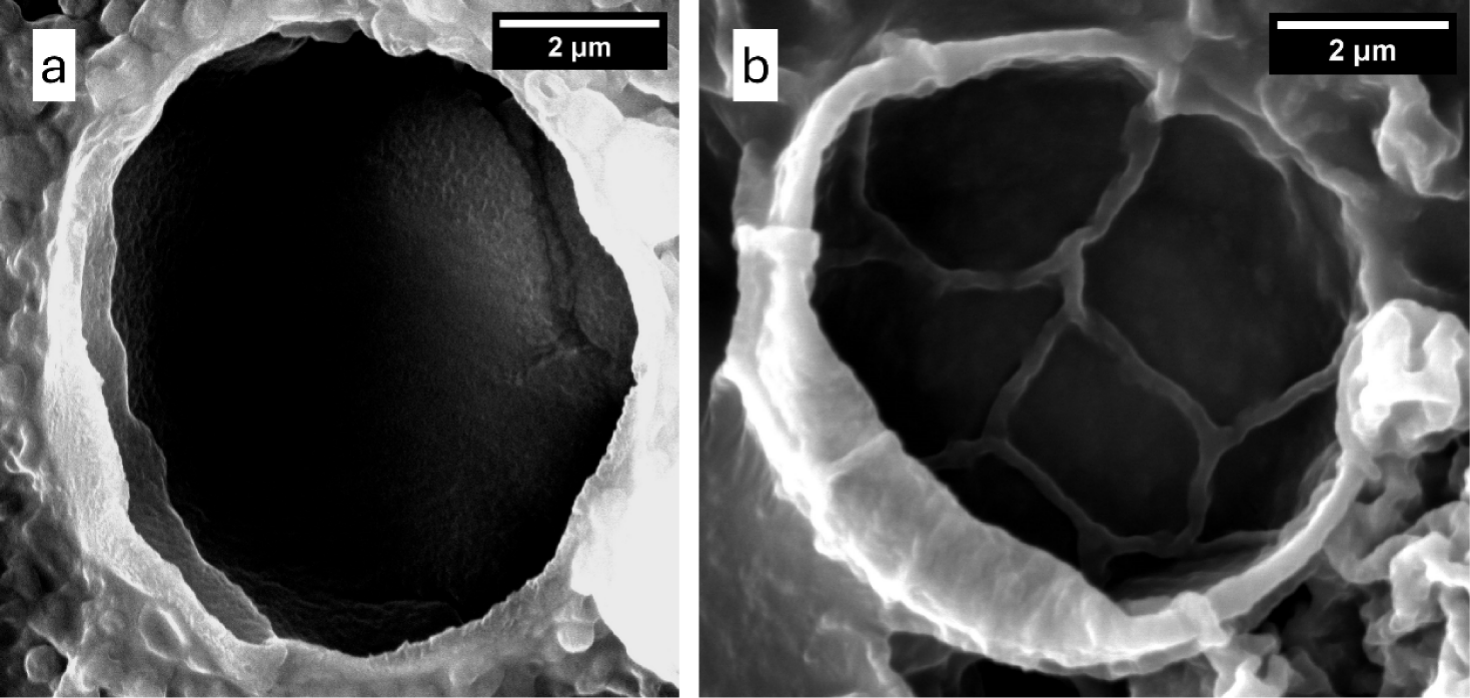
(a) and (b) Batch with 5% shell, depicting two broken microcapsules with a core–shell structure and an oil-hosting cavity.

### Encapsulation Efficiency and Payload

Table 2 summarizes the EE and payloads of all four batches. A high cinmethylin EE (∼99%) indicating a highly efficient encapsulation process, was observed for all four batches of microcapsules. A low water solubility (63 mg L^–1^)^24^ and a high *n*-octanol/water partition coefficient (Clog *p* = 4.6)^42^ for cinmethylin also contributes to the high EE values. A clear trend in the payload values was observed corresponding to the shell percentage, wherein the experimental payload roughly matched the theoretical cinmethylin payload for each batch. Practically, the PVA colloid used for emulsion stabilization is difficult to wash off completely and remains behind during the drying process prior to the payload experiment. Theoretically, PVA constitutes 3.8% of the total dry mass of the formulation. Therefore, a discrepancy in payload percentages (<3.8%) could be observed as in case of capsules with 5% shell.

**Table 2:** EE and Payload for Microcapsules^a^

Shell percentage (wt %)	EE (%)	Theoretical cinmethylin payload (%)	Experimental cinmethylin payload (%)
20	99.9 ± 0.1	80	79.2 ± 0.2
15	99.81 ± 0.04	85	84.4 ± 0.6
10	99.87 ± 0.03	90	89.6 ± 0.7
5	99.80 ± 0.01	95	92.4 ± 0.1

a Values after ± represent the standard error of the mean of duplicates.

### Polyurea Microcapsules

Polyurea microcapsules—with cinmethylin as the core—provided by BASF SE, were procured and analyzed. These polyurea microcapsules were synthesized using multifunctional isocyanates and amines leading to a cross-linked polyurea shell. Figure 7 shows the optical image of the capsule dispersion and the PSD for these polyurea microcapsules. As compared to the bis-urea microcapsules (Figure 4, *d*[3,2] = 2 μm) the polyurea microcapsules were larger in size (*d*[3,2] = 3.4 μm).

**Figure 7:**
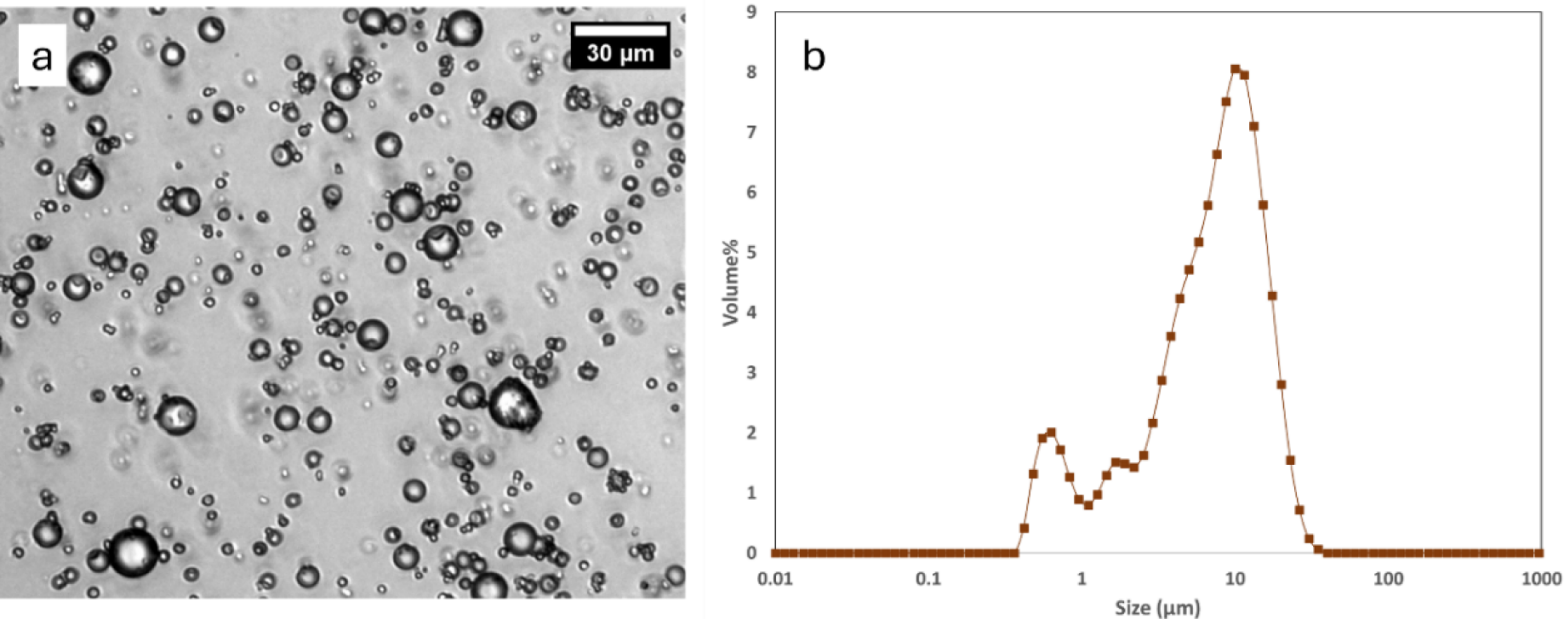
Polyurea microcapsules encapsulating cinmethylin (a) Optical image of the microcapsule dispersion (b) PSD.

Figure 8 shows the SEM images of these polyurea microcapsules. A clear difference in shell morphology was observed between these polyurea microcapsules and the bis-urea microcapsules. The polyurea microcapsules had a smooth exterior morphology, characteristic of the polymeric film which forms the shell. In contrast, the supramolecular shell of the bis-urea microcapsules has a rough appearance (Figure 5). Also, like the bis-urea microcapsules (Figure 5c,d), some of the polyurea microcapsules appeared to be deformed at the edges and damaged in the SEM chamber (Figure 8b) due to the high vacuum and electron beam.

**Figure 8:**
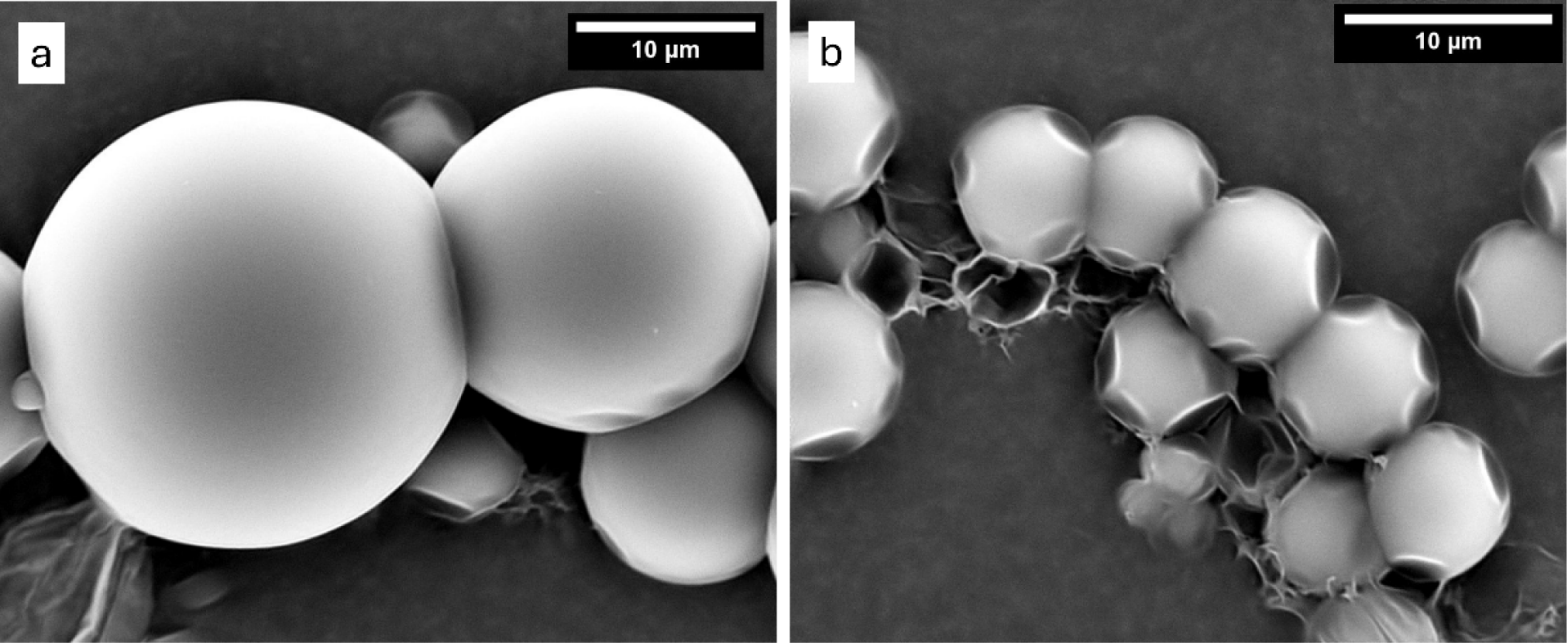
(a) and (b) SEM images (with different magnifications) of the polyurea microcapsules.

### Mechanical Strength

Figure 9 shows the typical curves for force vs distance traveled by the probe when a capsule is compressed for the polyurea and bis-urea microcapsules. From 0 to point *i,* the probe travels down toward the capsule (i.e., no contact). At *i,* the probe contacts the capsule and starts to compress it. Correspondingly, the force acting on the probe increases as it compresses the capsule until point *ii,* where the capsule ruptures or cracks and there is a slight drop in force. At point *iii,* the probe touches the glass slide which leads to a sharp increase in force acting on the probe. For all four batches of bis-urea microcapsules, the rupture force falls only slightly at the rupture point (*ii*, Figure 9a–d), which is distinct from the polyurea capsules (point *ii*, Figure 9e), where there is a significant drop in force at the rupture point, and is characteristic of a sudden “burst” at rupture. This difference in rupture behavior shows that the bis-urea microcapsules do not “burst” like the polymeric microcapsules completely, but “crack” leaving behind residual shell debris. Line segment (*ii–iii,* Figure 9a–d) represents the probe compressing this debris. All four bis-urea microcapsule batches showed a similar behavior showcasing the rigidity of the supramolecular shell as compared to the polyurea shell material.

**Figure 9:**
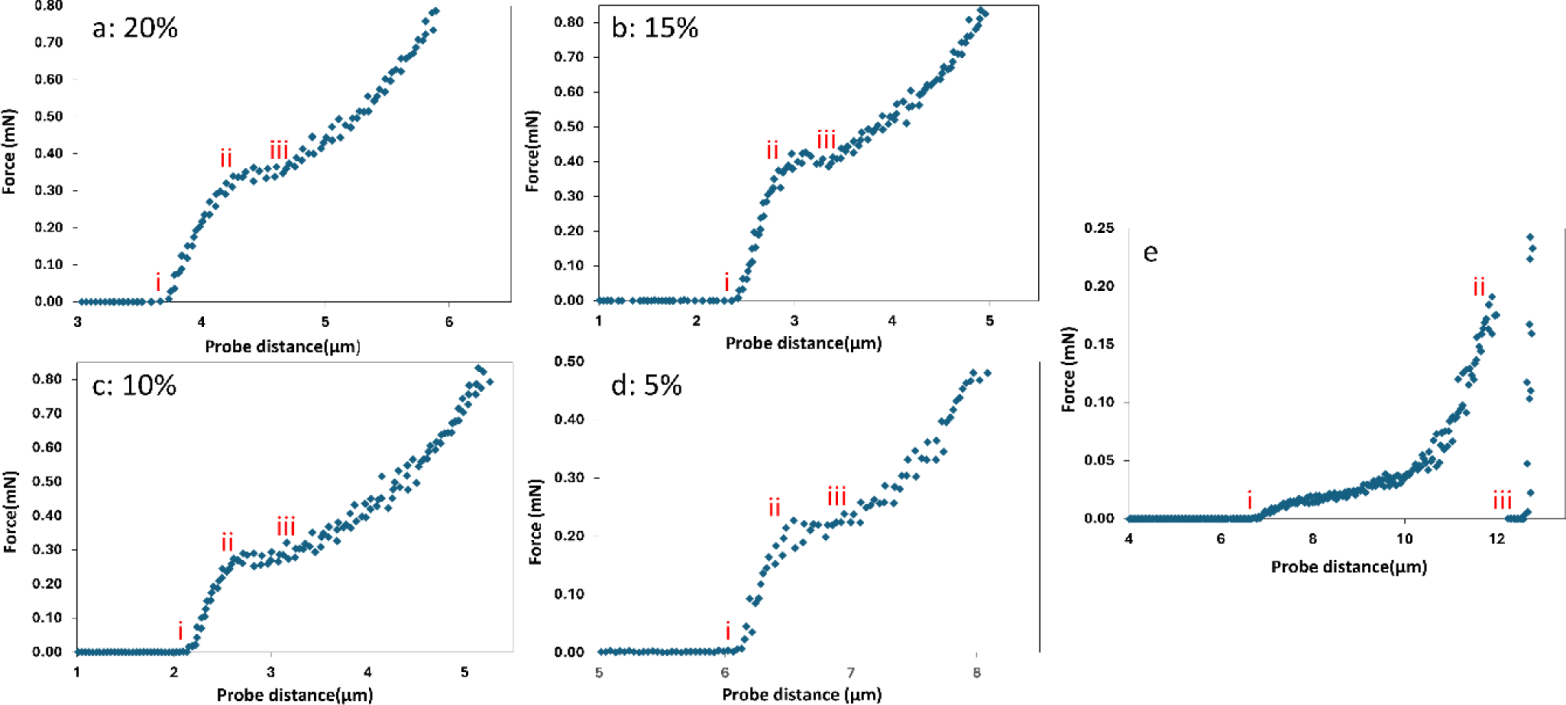
Force vs distance traveled by probe during compression of a single microcapsule with shell percentage (a) 20% (b) 15% (c) 10% (d) 5% and (e) polyurea microcapsules.

Table 3 summarizes the mechanical strength parameters for all the batches of microcapsules. Nominal rupture stress did not decrease significantly when the shell percentage was lowered from 20% to 15%. However, a statistically significant decrease in nominal rupture stress (∼3 MPa) was observed in microcapsules with 10% shell and a further reduction (∼4 MPa) was observed in capsules with 5% shell.

**Table 3:** Mechanical Strength Parameters of the Microcapsules^a^

Shell percentage	20%	15%	10%	5%	1.7% (polyurea)
Mean diameter, (μm)	6.7 ± 0.3	5.9 ± 0.3	6.3 ± 0.2	6.7 ± 0.2	12.7 ± 0.4
Displacement at rupture (μm)	1.0 ± 0.1	0.7 ± 0.1	0.7 ± 0.1	0.8 ± 0.1	4.9 ± 0.5
Rupture force (mN)	0.42 ± 0.04	0.34 ± 0.02	0.28 ± 0.02	0.20 ± 0.02	0.21 ± 0.04
Deformation at rupture (%)	14.1 ± 1.5	12.3 ± 1.2	12.1 ± 1.0	11.8 ± 1.0	38.2 ± 3.6
Nominal rupture stress (MPa)	11.7 ± 0.6	13.4 ± 1.1	9.2 ± 0.5	5.6 ± 0.4	1.6 ± 0.2
Rupture tension (μN/μm)	60.1 ± 3.2	58.2 ± 2.9	44.4 ± 2.1	29.4 ± 2.8	15.9 ± 2.5
Toughness (MPa)	0.91 ± 0.16	0.83 ± 0.17	0.57 ± 0.10	0.31 ± 0.05	0.17 ± 0.03

a Numbers after ± represent the standard error of the mean of 30 capsules.

The rupture tension is a size independent parameter, which is defined by the ratio of the rupture force to the diameter of the microcapsule. Therefore, microcapsules of different sizes can be compared using their rupture tensions. All four batches of bis-urea microcapsules exhibited higher rupture tensions than the polyurea microcapsules. The polyurea microcapsules were reported to have a cinmethylin payload of 98.3% by the manufacturer which implied that the shell material accounted for only 1.7% of the capsule composition. This could explain the relatively low rupture tension observed. As the shell percentage decreased progressively from 20% to 5%, a corresponding decrease in rupture tension was noted, further supporting the influence of shell thickness on the mechanical strength of the capsules. Additionally, the polyurea microcapsules exhibited a significantly larger deformation at rupture (38.2 ± 3.6%) as compared to all the bis-urea microcapsules (∼12%). This increase in deformation can be attributed to the flexibility of the polymeric shell which allows it to deform more than the supramolecular bis-urea shell before rupturing.

Bhutkar et al. tested bis-urea microcapsules using the same micromanipulation technique.^32^ The bis-urea molecule used for the self-assembly had an aliphatic six-carbon straight chain structure. The corresponding microcapsules with a 68% payload had a rupture tension of 23 ± 2 μN/μm. Inclusion of aromatic rings is known to increase the rigidity and mechanical strength of materials.^43,44^ In addition to the hydrogen bonds from the urea linkages, the aromatic rings enable intermolecular π–π interactions which will aid and strengthen the supramolecular self-assembly.^45^ Consequently, the microcapsules formed in the present study, using aromatic bis-urea molecules (Scheme 1) exhibited ca. 160% increase in rupture tension (60.1 ± 3.2 μN/μm for 20% shell). Additionally, the rupture tension of bis-urea microcapsules could be tuned by changing the shell percentage (29.4 to 60.1 μN/μm). These rupture tension values are not limited to microcapsules encapsulating agrochemical active ingredients but are also similar to commercial polymeric microcapsules used in consumer care products (range of ∼7 to 111 μN/μm),^46^ demonstrating that the replacement of covalent cross-linking with noncovalent supramolecular bonding does not compromise microcapsule strength.

### Accelerated Isothermal Release of Cinmethylin

The microcapsules were stored as aqueous dispersions in PVA solutions, and both the payload and EE remained unchanged over 12 months, indirectly indicating long-term stability under aqueous conditions, a requirement for many agrochemical formulations.

At the time of application, the microcapsules are sprayed onto the field, and the herbicide is subsequently released under dry conditions. To simulate this scenario, an accelerated thermal release test was conducted using TGA to evaluate the effect of encapsulation on cinmethylin evaporation. Dry microcapsules were maintained at 130 °C in the TGA furnace and the corresponding loss of cinmethylin was recorded for 3 h. The TGA and DSC curves for the microcapsule shell material are included in (Figure S5). From the figure, the melting point of the empty shell material was estimated to be 250 °C, and therefore the microcapsule shell was considered to be thermally stable during the release test. Figure 10 represents the thermal release in terms of mass %. During the control experiment, almost all the unencapsulated cinmethylin (99%) evaporated in 3h (Figure 10a). Encapsulation significantly reduced the mass loss, wherein only 38% cinmethylin was lost from microcapsules with 5% shell in 3 h. As the shell percentage increased to 10%, the mass loss in 3 h reduced further to approximately 15%, while the microcapsules with 15% shell limited the cinmethylin loss to 10%. However, a further increase in shell percentage to 20% did not lead to significant improvement in barrier properties.

**Figure 10:**
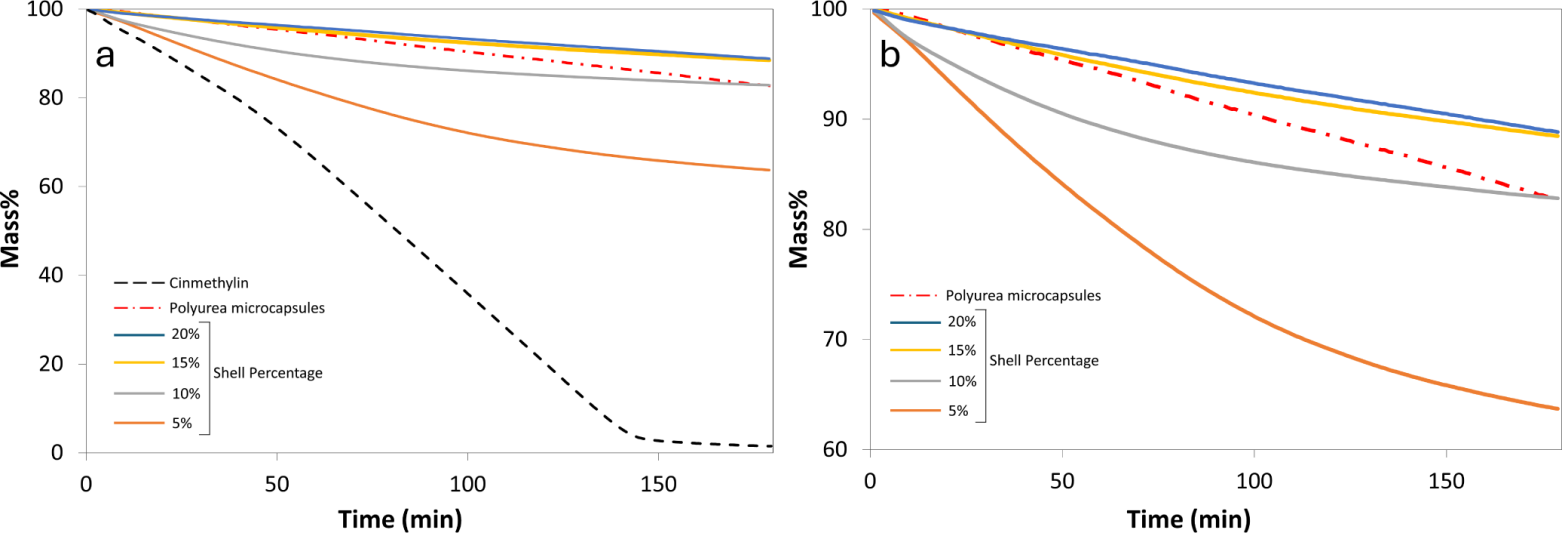
Mass loss of cinmethylin at 130 °C. (a) All release profiles in comparison with unencapsulated cinmethylin. (b) Release profiles of microcapsules rescaled for better resolution.

Microcapsules with 10% shell matched the performance of the polyurea microcapsules in terms of mass loss after 3 h. However, a clear difference in the nature of release profiles is observed in Figure 10b. Capsules with 20% and 15% shell, as well as the polyurea microcapsules demonstrated an almost linear loss in cinmethylin. At a constant temperature, the concentration gradient of cinmethylin, defined as the difference in concentration inside and outside the microcapsules is the major driving force for the release. Initially, this driving force is high (can be assumed to be constant) when the amount of cinmethylin released is low. The release rate consequently remains constant and follows zero order kinetics. As the amount of cinmethylin released increases, the concentration of cinmethylin inside the microcapsules reduces and consequently the concentration gradient also decreases. This causes the release rate to drop, which gives rise to an exponential release profile, as in case of microcapsules with 10% and 5% shell. Similar models explaining the isothermal release of volatile actives from microcapsules have been reported previously.^47,48^

Extending the release tests beyond 3 h may reveal a transition from near-linear to nonlinear release profiles for microcapsules with 15% and 20% shell content as more cinmethylin is released. Nevertheless, the primary aim of the current study was to establish a comparative framework to evaluate the effect of encapsulation on cinmethylin release, particularly benchmarking the performance of supramolecular bis-urea microcapsules against conventional polyurea microcapsules. The results presented successfully achieved this aim, and future work will include detailed long-term studies to further elucidate the release mechanisms. Most importantly, this thermal release experiment proved that the bis-urea microcapsules could perform at par with the conventional polyurea microcapsules. Moreover, insufficient release of cinmethylin at the site of action was identified as a problem in some of the commercial polymeric microcapsules and the shell composition had to be tuned accordingly.^27^ The thermal release data demonstrates that cinmethylin release from bis-urea microcapsules can be adjusted easily by regulating the shell percentage and the performance of the bis-urea microcapsules could be tuned as per the final application.

## Conclusions

In this work, microplastic-free cinmethylin microcapsules were successfully fabricated using supramolecular self-assembly of bis-urea molecules in a one-pot *in situ* process. Mass spectroscopy, ^1^H NMR and FT-IR spectroscopy proved the absence of any polyurea in the shell material confirming that the microcapsules were microplastic-free. Four batches of microcapsules with varying shell percentage were fabricated, all showing excellent EE (>99%). The inclusion of an aromatic ring in the bis-urea molecule significantly increased the mechanical strength (>160% increase in rupture tension) of the supramolecular shell compared to aliphatic bis-urea microcapsules reported previously.^32^

Compared to conventional polyurea microcapsules, all four batches of bis-urea microcapsules demonstrated at least double rupture tension. Accelerated isothermal release tests at 130 °C, showed that encapsulation reduced the cinmethylin evaporation from 99% to as low as 10% after 3 h. Microcapsules with 10%, 15%, and 20% shell had a release performance comparable to the polyurea microcapsules. The release rate can be tuned by adjusting the shell percentage indicating that the barrier properties of the supramolecular shell can be controlled easily, broadening the applicability of these bis-urea microcapsules to encapsulate various AI beyond agrochemicals. Furthermore, standard interfacial polymerization equipment can be used for fabricating these bis-urea microcapsules without modification, making them easy to manufacture in production plant and viable for commercialization.

## Supplementary Material



## Notes

Mass spectroscopy, ^1^H NMR spectroscopy, and the TGA and DSC (microcapsule shell) data (PDF)

## References

[1] Yılmaz H., Enginar H., Çifci C. (2021). Microencapsulation of Pendimethalin
with Polyurethane-Urea and Determination of Its Stability. J. Taibah Univ. Sci..

[2] Koli, P. ; Bhardwaj, N. R. ; Mahawer, S. K. Chapter 4 - Agrochemicals: Harmful and Beneficial Effects of Climate Changing Scenarios. In Climate Change and Agricultural Ecosystems, Choudhary, K. K. ; Kumar, A. ; Singh, A. K. , Eds.; Woodhead Publishing, 2019, pp. 65–94. 10.1016/B978-0-12-816483-9.00004-9.

[3] Devi P. I., Manjula M., Bhavani R. V. (2022). Agrochemicals, Environment,
and Human
Health. Annu. Rev. Environ. Res..

[4] Bourguet, D. ; Guillemaud, T. The Hidden and External Costs of Pesticide Use. In Sustainable Agriculture Reviews, Lichtfouse, E. , Ed.; Springer International Publishing: Cham, 2016, pp. 35–120. 10.1007/978-3-319-26777-7_2

[5] Udeigwe T. K., Teboh J. M., Eze P. N., Hashem Stietiya M., Kumar V., Hendrix J., Mascagni H. J., Ying T., Kandakji T. (2015). Implications of Leading Crop Production Practices on
Environmental Quality and Human Health. J. Environ.
Manage..

[6] Akelah A. (1996). Novel Utilizations
of Conventional Agrochemicals by Controlled Release Formulations. Mater. Sci. Eng., C.

[7] Pang L., Gao Z., Feng H., Wang S., Wang Q. (2019). Cellulose Based Materials
for Controlled Release Formulations of Agrochemicals: A Review of
Modifications and Applications. J. Controlled
Release.

[8] Hack B., Egger H., Uhlemann J., Henriet M., Wirth W., Vermeer A. W. P., Duff D. G. (2012). Advanced Agrochemical Formulations
through Encapsulation Strategies?. Chem. Ing.
Tech..

[9] Andrade B., Song Z., Li J., Zimmerman S. C., Cheng J., Moore J. S., Harris K., Katz J. S. (2015). New Frontiers
for Encapsulation in the Chemical Industry. ACS Appl. Mater. Interfaces.

[10] Zhang Y., Rochefort D. (2012). Characterisation
and Applications of Microcapsules
Obtained by Interfacial Polycondensation. J.
Microencapsul..

[11] Perignon C., Ongmayeb G., Neufeld R., Frere Y., Poncelet D. (2015). Microencapsulation
by Interfacial Polymerisation: Membrane Formation and Structure. J. Microencapsul..

[12] Patchan M. W., Fuller B. W., Baird L. M., Gong P. K., Walter E. C., Vidmar B. J., Kyei I., Xia Z., Benkoski J. J. (2015). Robust
Composite-Shell Microcapsules via Pickering Emulsification. ACS Appl. Mater. Interfaces.

[13] Yang Y., Wei Z., Wang C., Tong Z. (2013). Versatile Fabrication of Nanocomposite
Microcapsules with Controlled Shell Thickness and Low Permeability. ACS Appl. Mater. Interfaces.

[14] Wong, L. W. ; Fahimizadeh, M. ; Tan, J. B. L. ; Pasbakhsh, P. Chapter 11 - Polyurea Microcapsules as Effective Carriers for Biomedical and Agricultural Applications, Pasbakhsh, P. ; Mohotti, D. ; Palaniandy, K. ; Auckloo, S. A. B. , Eds.; Elsevier, 2023, pp. 191–202. 10.1016/B978-0-323-99450-7.00019-8.

[15] Tylkowski B., Olkiewicz M., Montane X., Nogalska A., Haponska M., Montornes J. M., Kowalska J., Malusá E. (2020). 11. Encapsulation
Technologies in Agriculture. Microencapsulation.

[16] Yu F., Wang Y., Zhao Y., Chou J., Li X. (2021). Preparation
of Polyurea Microcapsules by Interfacial Polymerization of Isocyanate
and Chitosan Oligosaccharide. Materials.

[17] Rao J., Chandrani A. N., Powar A., Chandra S. (2023). Preparation of Microcapsule
Suspension of Herbicide Oxyfluorfen Polyurea and Its Effects on Phytotoxicity
on Rice Crop. J. Dispers. Sci. Technol..

[18] Bruyninckx K., Dusselier M. (2019). Sustainable Chemistry Considerations for the Encapsulation
of Volatile Compounds in Laundry-Type Applications. ACS Sustainable Chem. Eng..

[19] Woźniak-Budych M., Staszak K., Wieszczycka K., Bajek A., Staszak M., Roszkowski S., Giamberini M., Tylkowski B. (2024). Microplastic
Label in Microencapsulation Field – Consequence of Shell Material
Selection. J. Hazard. Mater..

[20] Lobel B. T., Baiocco D., Al-Sharabi M., Routh A. F., Zhang Z., Cayre O. J. (2024). Current Challenges
in Microcapsule Designs and Microencapsulation
Processes: A Review. ACS Appl. Mater. Interfaces.

[21] Lamichhane G., Acharya A., Marahatha R., Modi B., Paudel R., Adhikari A., Raut B. K., Aryal S., Parajuli N. (2023). Microplastics
in Environment: Global Concern, Challenges, and Controlling Measures. Int. J. Environ. Sci. Technol..

[22] Liu L., Wang Z., Ye Y., Qi K. (2023). Effects of Agricultural
Land Types on Microplastic Abundance: A Nationwide Meta-Analysis in
China. Sci. Total Environ..

[23] ECHA. Amending Annex XVII to Regulation (EC) No 1907/2006 of the European Parliament and of the Council concerning the Registration, Evaluation, Authorisation and Restriction of Chemicals (REACH) as regards synthetic polymer microparticles. https://single-market-economy.ec.europa.eu/publications/commission-regulation-eu-amending-reach-regulation-regards-synthetic-polymer-microparticles_en (accessed January 20, 2024).

[24] Grayson B. T., Williams K. S., Freehauf P. A., Pease R. R., Ziesel W. T., Sereno R. L., Reinsfelder R. E. (1987). The Physical and Chemical Properties
of the Herbicide Cinmethylin (SD 95481). Pestic.
Sci..

[25] Xu H., Leng Q., Su W., Sun L., Cheng J., Wu R. (2024). Herbicidal Activity of Cinmethylin against Grass Weeds and Its Safety
for Use with Different Wheat Varieties. Agronomy.

[26] Comont D., Crook L., Hull R., Sievernich B., Kevis S., Neve P. (2024). The Role of Interspecific
Variability
and Herbicide Pre-Adaptation in the Cinmethylin Response Of. Pest Manage. Sci..

[27] Kolb, K. ; Nolte, M. ; Gregori, W. ; Schmitt, M. ; Franz, D. ; Kraus, H. Composition Comprising Polyurethane Microcapsules Comprising Cinmethylin. WO 2,018,104,118 A1, 2018.

[28] Urch, H. ; Schmitt, M. ; Kolb, K. ; Franz, D. ; Klimov, E. ; Kraus, H. Composition Comprising Cinmethylin-Containing Microparticles and a Further Herbicide. WO 2,018,130,588 A1, 2018.

[29] Burakowska-Meise, E. ; Mecfel-Marczewski, J. ; Bratz, M. ; Denuell, W. ; Bowe, S. ; Repage, R. ; Frihauf, J. Anionic Polyvinyl Alcohol Copolymer as Protective Colloid for Pesticidal Polyurea Microcapsules. WO 2,015,165,834 A1, 2015.

[30] Beestman, G. B. Microcapsules Formulations of Agricultural Chemicals. WO 1,994,013,139 A1, 1994.

[31] Palanichamy, K. Storage-Stable Compositions Including Bixlozone and Beflubutamid. WO 2,024/,073,019 A1, 2024.

[32] Bhutkar S. P., Millard P.-E., Preece J. A., Zhang Z. (2024). Microplastic-Free Microcapsules
Using Supramolecular Self-Assembly of Bis-Urea Molecules at an Emulsion
Interface. Langmuir.

[33] Zhang Z., Saunders R., Thomas C. R. (1999). Mechanical
Strength of Single Microcapsules
Determined by a Novel Micromanipulation Technique. J. Microencapsul..

[34] Han H., Li S., Zhu X., Jiang X., Kong X. Z. (2014). One Step Preparation
of Porous Polyurea by Reaction of Toluene Diisocyanate with Water
and Its Characterization. RSC Adv..

[35] Ning L., De-Ning W., Sheng-Kang Y. (1996). Hydrogen Bonding
between Urethane
and Urea: Band Assignment for the Carbonyl Region of FTi.r. Spectrum. Polymer.

[36] Sravan B., Kamalakar K., Karuna M. S. L., Palanisamy A. (2014). Studies on
Organogelation of Self Assembling Bis Urea Type Low Molecular Weight
Molecules. J. Sol-Gel Sci. Technol..

[37] Boileau S., Bouteiller L., Lauprêtre F., Lortie F. (2000). Soluble Supramolecular
Polymers Based on Urea Compounds. New J. Chem..

[38] Ocepek M., Zabret J., Kecelj J., Venturini P., Golob J. (2015). Monitoring of Polyurethane Dispersions
after the Synthesis. Mater. Technol..

[39] Pannone M. C., Macosko C. W. (1987). Kinetics of Isocyanate
Amine Reactions. J. Appl. Polym. Sci..

[40] Urch, H. ; Franz, D. ; Nolte, M. ; Kraemer, G. ; Michel, A. ; Kolb, K. Microcapsules Comprising Cinmethylin in the Core and a Polyurea Derived from Diphenylmethane Diisocyanate or an Oligomer Thereof. WO 2,018,130,589 A1, 2018.

[41] Atiemo-Obeng, V. A. ; Calabrese, R. V. Rotor–Stator Mixing Devices. In Handbook of Industrial Mixing; John Wiley & Sons, Ltd, 2003, pp. 479–505. 10.1002/0471451452.ch8.

[42] Hsu F. C., Marxmiller R. L., Yang A. Y. S. (1990). Study of Root Uptake and Xylem Translocation
of Cinmethylin and Related Compounds in Detopped Soybean Roots Using
a Pressure Chamber Technique. Plant Physiol..

[43] Mengdi C., Wei L., Changping Y., Suli X., Su J., Liping S. (2021). The Influence
of Molecular Structure on the Thermal Properties and Processability
of Nitrile-Based Resin Molecules. J. Phys.:
Conf. Ser..

[44] Mohanty, A. D. ; Bae, C. Chapter One - Transition Metal-Catalyzed Functionalization of Polyolefins Containing CC, CC, and CH Bonds. In Advances in Organometallic Chemistry, Pérez, P. J. , Ed.; Academic Press, 2015; Vol. 64, pp. 1–39. 10.1016/bs.adomc.2015.08.002.

[45] Melia K., Greenland B. W., Hermida-Merino D., Hart L. R., Hamley I. W., Colquhoun H. M., Slark A. T., Hayes W. (2018). Self-Assembling Unsymmetrical
Bis-Ureas. React. Funct. Polym..

[46] Smets, J. ; Sands, P. D. ; Guinebretiere, S. J. ; Pintens, A. ; Dihora, J. O. Benefit Agent Containing Delivery Particle. WO 2,008,066,773 A2, 2008.

[47] Yeh K.-W., Chang C. P., Yamamoto T., Dobashi T. (2011). Release Model of Alginate
Microcapsules Containing Volatile Tea-Tree Oil. Colloids Surf., A.

[48] Foroutani M., Jahanmardi R., Atai M., Nodehi A. (2024). Temperature-Sensitive
Fragrance-Releasing Polymeric Microcapsules. Eur. Polym. J..

