# Supplementary material for: Self-Assembled Microplastic-Free Microcapsules Using Aromatic Bis-Ureas with Improved Strength and Tunable Barrier Properties for Encapsulating Cinmethylin
Source: ACS Appl Mater Interfaces. 2025 May 14;17(21):31522–33. doi: 10.1021/acsami.5c06238 (PMC12123565; doi:10.1021/acsami.5c06238)
Supplement: Supplementary file 1 [file am5c06238_si_001.pdf]

Supporting Information

Self-assembled microplastic-free microcapsules using aromatic bis-ureas with improved strength and tunable barrier properties for encapsulating cinmethylin

Siddhant Pravin Bhutkar[a], Pierre-Eric Millard[c], Henning Urch[d], Jon A. Preece[b] and Zhibing Zhang[a,]*

[a]School of Chemical Engineering, University of Birmingham, Birmingham B15 2TT, UK

[b]School of Chemistry, University of Birmingham, Birmingham B15 2TT, UK

[c]BASF SE, 67056 Ludwigshafen am Rhein, Germany

[d]BASF SE, 67117 Limburgerhof, Germany

*Corresponding author, email: z.zhang@bham.ac.uk

Section S1: Mass Spectroscopy

The mass spectra of the microcapsule shell material and the pure bis-urea standard were recorded using a Waters Xevo G2 mass spectrometer equipped with an electrospray ionization (ESI) source operated in direct infusion mode. Methanol was used as a solvent for both samples and data was acquired in time-of-flight (TOF) mode, operating in positive ionization mode upto 2500 m/z. **Figure S1** shows the spectra for both samples, which exhibit similar peaks, including a prominent peak at m/z 373 corresponding to the desired bis-urea structure (**Scheme 1,** 373 g mol^{-1}). Additional peaks can be attributed to various factors: eg. cationic adducts (eg. M + Na at m/z 395), fragmentation products (eg. m/z 291) or other impurities intrinsic to the raw materials or solvent as evidenced by identical peaks observed for both the samples.

Notably, the peak at m/z 745 corresponds to the dimer of the bis-urea, likely formed due to supramolecular hydrogen bonding. Most importantly, the peak representing the undesired oligomer depicted in **Scheme 2** (ca. 520 g mol^{-1}) does not appear in the spectrum for the microcapsule shell. Furthermore, no peaks corresponding to higher molecular weight oligomers were observed in the spectrum for the microcapsule shell (**Figure S1a**).

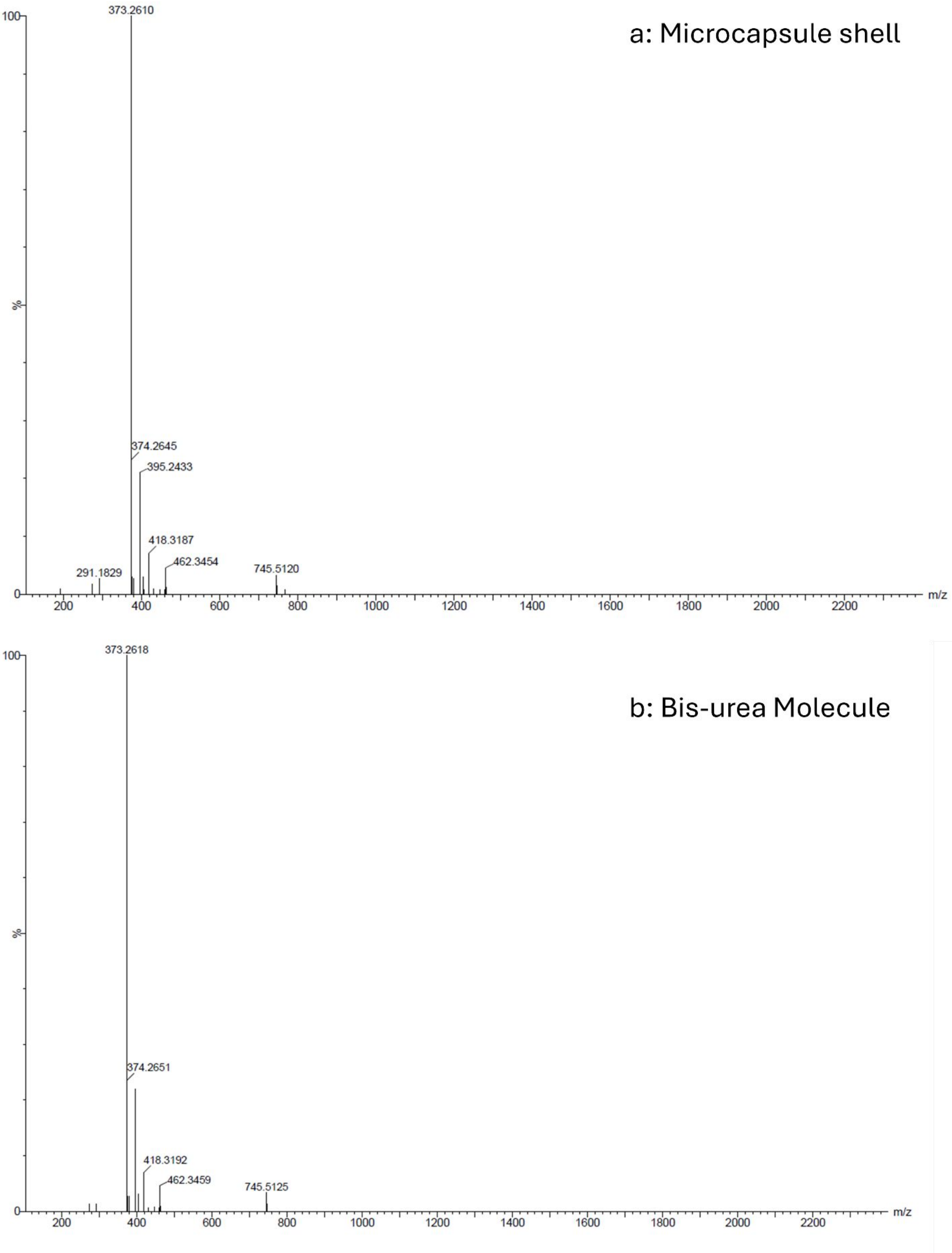

Figure S1: Mass spectrum for (a) Microcapsule shell (b) Bis-urea molecule

Section S2. ^{1}H -NMR spectrum

The ^{1}H-NMR spectrum for the bis-urea molecule and the shell material was recorded in deuterated dimethyl sulfoxide (DMSO-d6) using the Avance Neo Spectrometer (Bruker, USA) operating at 400 MHz and analysed using MestReNova (Version 14.2.1). **Figure S2** shows a complete overlap of the two ^{1}H-NMR spectra. The DMSO-d6 peak at 2.50 ppm is used as a reference and the strong peak at 3.33 ppm is associated with water.

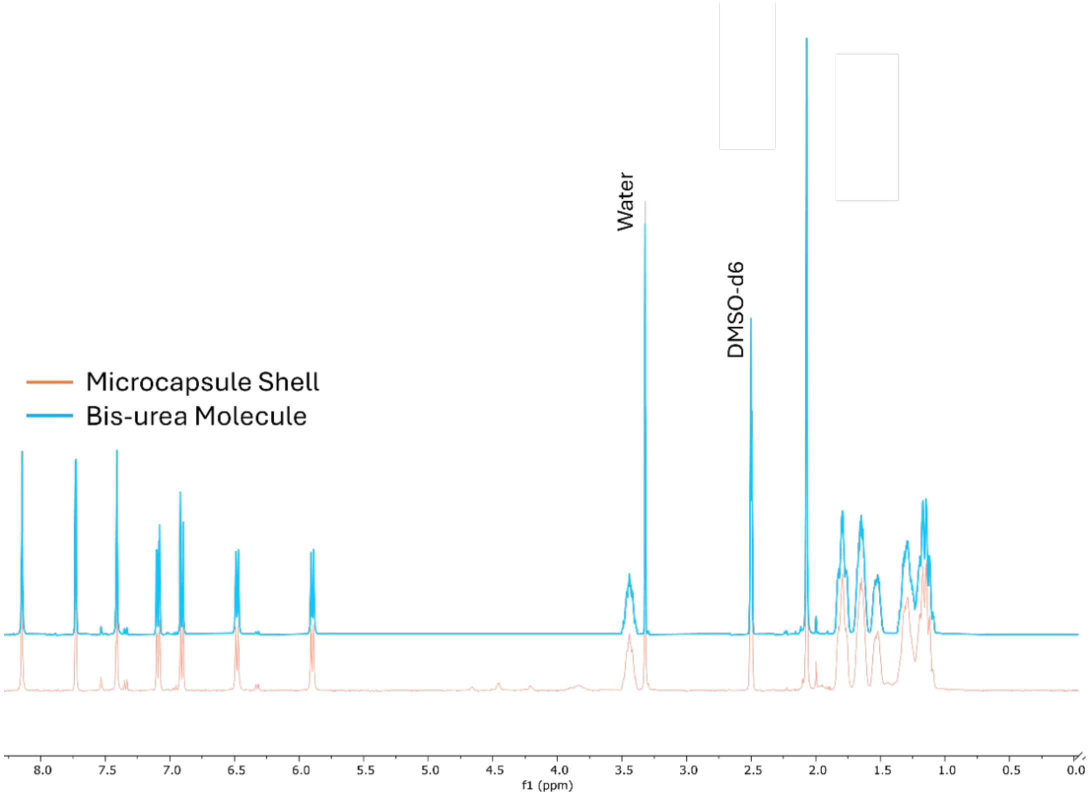

Figure S2: ^{1}H-NMR spectra for the microcapsule shell and bis-urea molecule.

Overall, the strong overlap of both the spectra suggested that the microcapsule shell also had the same chemical composition as the pure bis-urea used as a standard. Nevertheless, weak signals associated to impurities were observed at roughly 7.53, 7.33, 6.33, 2.23 and 1.99 ppm. However, these peaks are also observed, at the same chemical shift, in the spectrum for the bis-urea molecule synthesized in organic conditions in the absence of water. Therefore, these weak peaks are most likely associated with the impurities inherently present in the raw materials and not with the undesirable side products arising from -NCO hydrolysis represented in **Scheme 2**.

Additional peaks are observed only with the microcapsule shell at 4.66, 4.45, 4.21 and 3.83 ppm. These signals are associated to the residual PVA used for emulsifying the oil phase during microcapsule synthesis, which is difficult to remove completely during the washing. The first

three peaks are attributed to the -OH proton and the last peak is assigned to the -CH. Korbag and Mohamed Saleh have reported a similar spectrum for pure PVA of the same grade used in this study.[1] All the signals mentioned hitherto were not considered in the assignments and integration described in the following paragraphs for the microcapsule shell.

Figure S3 shows the spectrum for the microcapsule shell highlighting the region attributing to protons in the aromatic ring and the urea linkage. All four -NH protons appear as separate peaks. The two -NH protons closer to the aromatic ring (9, 16) appear as singlets at 8.15 and 7.41 ppm. The other two -NH protons closer to cyclohexyl rings (7, 18) appear at 6.48 and 5.90 ppm as doublets due to the coupling with the -CH in the ring. The aromatic proton at position

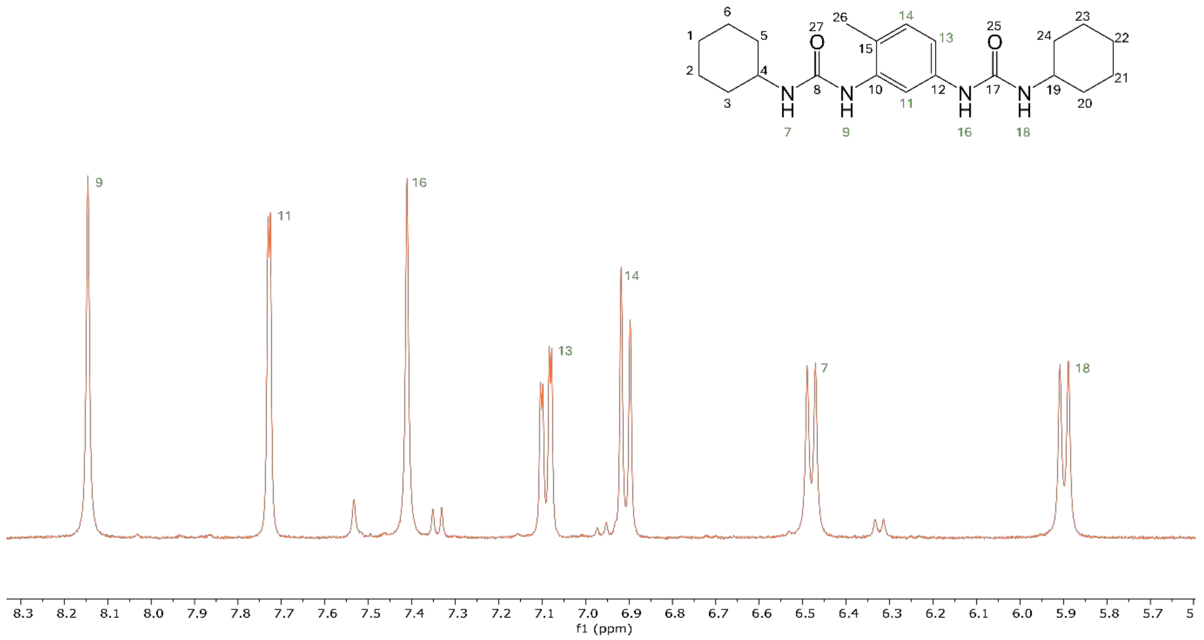

Figure S3: ^{1}H-NMR spectrum of the microcapsule shell for the aromatic protons.

11 appears as a doublet at 7.73 ppm coupling with the proton at position 13. The proton at position 13 itself couples with the other two aromatic protons and appears as a doublet of doublets at 7.09 ppm. The proton at position 14 appears as a doublet at 6.90 ppm. Simic et al. observed similar chemical shifts for symmetrical bis-ureas synthesized using TDI.[2]

Figure S4 shows the aliphatic protons of the molecule. The -CH_3 (26) appears as a strong singlet at 2.09 ppm. The two -CH protons (4,19) both coupled with four other protons in the cyclohexyl ring appear at 3.44 ppm. The -CH_2 protons of the cyclohexyl rings appear as multiplets from 1-1.90 ppm.

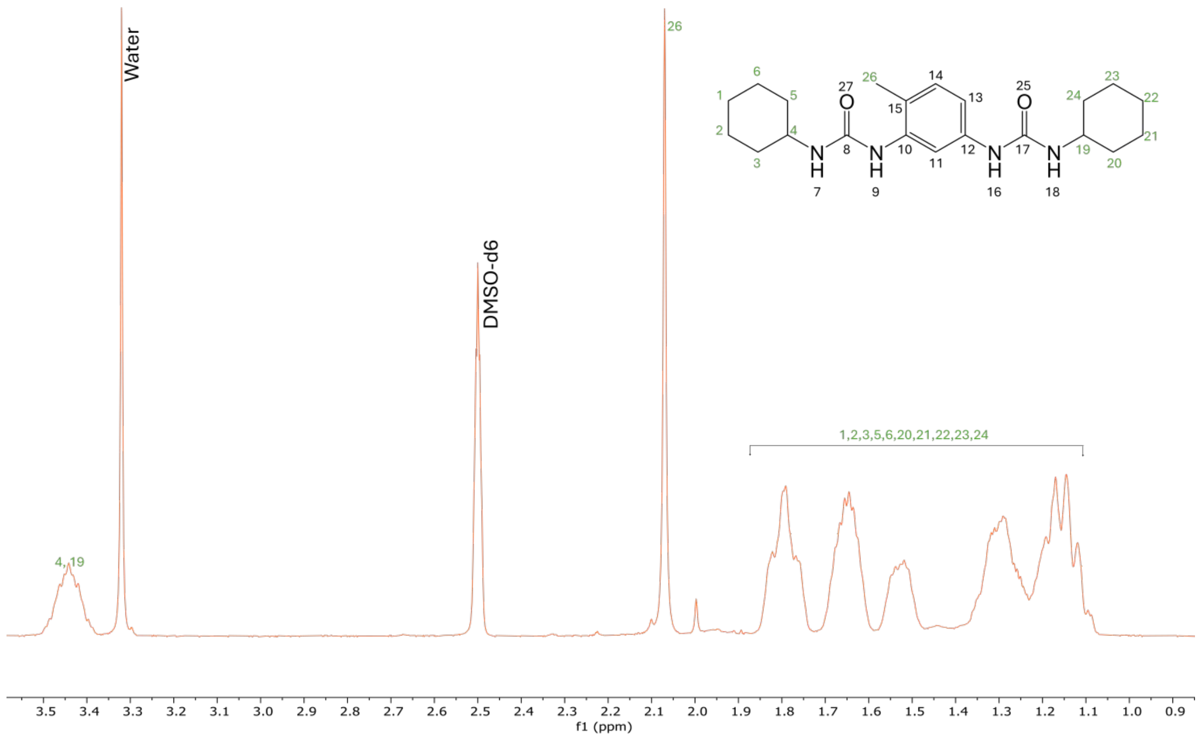

Figure S4: ^{1}H-NMR spectrum of the microcapsule shell for the aliphatic protons

Microcapsule shell: ^{1}H NMR (400 MHz, DMSO): δ (ppm) = 8.15 (s, 1H), 7.73 (d, *J* = 2.3 Hz, 1H), 7.41 (s, 1H), 7.09 (dd, *J* = 8.2, 2.3 Hz, 1H), 6.91 (d, *J* = 8.4 Hz, 1H), 6.48 (d, *J* = 7.6 Hz, 1H), 5.90 (d, *J* = 7.8 Hz, 1H), 3.52 – 3.37 (m, 2H), 2.07 (s, 3H), 1.87 – 1.72 (m, 4H), 1.72 – 1.59 (m, 4H), 1.53 (d, *J* = 13.3 Hz, 2H), 1.37 – 1.07 (m, 10H).

Bis-urea molecule: ^{1}H NMR (400 MHz, DMSO): δ (ppm) = 8.14 (s, 1H), 7.73 (d, *J* = 2.2 Hz, 1H), 7.41 (s, 1H), 7.09 (dd, *J* = 8.2, 2.3 Hz, 1H), 6.91 (d, *J* = 8.2 Hz, 1H), 6.48 (d, *J* = 7.7 Hz, 1H), 5.90 (d, *J* = 7.8 Hz, 1H), 3.52 – 3.38 (m, 2H), 2.07 (s, 3H), 1.87 – 1.72 (m, 4H), 1.65 (dp, *J* = 8.5, 5.1 Hz, 4H), 1.59 – 1.46 (m, 2H), 1.38 – 1.06 (m, 10H)

Comparing the shift and integration for both the spectra, a minimal deviation in shift for the aliphatic protons (1-1.90 ppm) is observed which is negligible. The total number of protons after integration is 32 which corresponds to the desired bis-urea molecule. Moreover, if the oligomers resulting from TDI hydrolysis were formed (Scheme 2), the signals from the -NH proton closer to the aromatic ring (9, 16 in Figure S3) would have integration >1H which does not happen. This indicates only one aromatic ring was present in the structure and we indeed have the desired bis-urea composition in the shell material without the presence of oligomers.

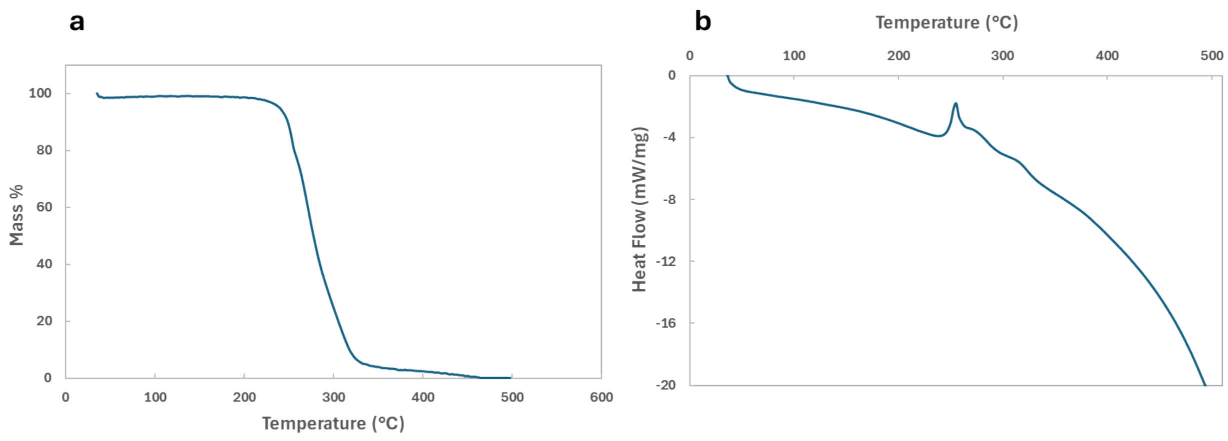

Figure S5: Curves obtained using Simultaneous Thermal Analysis (STA) for the microcapsule shell material (a) TGA; (b)DSC.

References

(1) Korbag, I.; Mohamed Saleh, S. Studies on the Formation of Intermolecular Interactions and Structural Characterization of Polyvinyl Alcohol/Lignin Film. *Int. J. Environ. Stud.* **2016**, *73* (2), 226–235. https://doi.org/10.1080/00207233.2016.1143700.

(2) Simic, V.; Bouteiller, L.; Jalabert, M. Highly Cooperative Formation of Bis-Urea Based Supramolecular Polymers. *J. Am. Chem. Soc.* **2003**, *125* (43), 13148–13154. https://doi.org/10.1021/ja037589x.
